# Efficient selective removal of uremic toxin precursor by olefin-linked covalent organic frameworks for nephropathy treatment

**DOI:** 10.1038/s41467-023-38427-3

**Published:** 2023-05-16

**Authors:** Jinxia Wei, Rui Li, Penghui Zhang, Haiqun Jin, Zhenjie Zhang, Yubo Li, Yao Chen

**Affiliations:** 1 School of Chinese Materia Medica, Tianjin University of Traditional Chinese Medicine, Tianjin, China; 2 State Key Laboratory of Medicinal Chemical Biology, College of Pharmacy, Nankai University, Tianjin, China; 3 College of Chemistry, Nankai University, Tianjin, China

**Keywords:** Organic molecules in materials science, Biomedical materials, Biomedical materials

## Abstract

Indoxyl sulfate is a protein-bound uremic toxin synthesized from indole that cannot be efficiently removed by the hemodialysis method and thus becomes a key risk factor for the progression of chronic kidney disease. Here, we develop a non-dialysis treatment strategy to fabricate an ultramicroporous olefin-linked covalent organic framework with high crystallinity in a green and scalable fashion for selectively removing the indoxyl sulfate precursor (i.e., indole) from the intestine. Various analyses show that the resulting material exhibits excellent gastrointestinal fluid stability, high adsorption efficiency, and good biocompatibility. Notably, it realizes the efficient and selective removal of indole from the intestine and significantly attenuates serum indoxyl sulfate level in vivo. More importantly, the selective removal efficacy of indole is substantially higher than that of the commercial adsorbent AST-120 used in the clinic. The present study opens up a new avenue to eliminate indoxyl sulfate by a non-dialysis strategy and further expands the in vivo applications of covalent organic frameworks.

## Abstract (web summary)

Indoxyl sulfate is a key risk factor in the progression of chronic kidney disease, but cannot be removed from the blood by hemodialysis. Here, the authors report the use of a covalent organic framework for the removal of indoxyl sulfate precursor from the intestine.

## Introduction

Chronic kidney disease (CKD) has become a global public health burden due to its increasing trends in the incidence and prevalence rates worldwide^1^. Without prompt treatment, CKD can develop into end-stage renal disease (ESRD) requiring dialysis or even kidney transplantation^2,3^. The accumulation of protein-bound uremic toxins (PBUTs), such as indoxyl sulfate (IS), is one of the major factors that cause the development and progression of CKD to ESRD^4–6^. IS is one of the representative PBUTs that is synthesized from indole generated by intestinal bacteria^7^, and it is of great challenge to remove IS via traditional dialysis treatment due to its high protein binding properties^8,9^. In addition, dialysis treatment often requires expensive medical expenses and repeated hospitalizations^10^. However, efficient and selective removal of indole, a precursor of IS, from the intestine becomes an effective measure to reduce serum IS level. Therefore, a non-dialysis strategy targeting the substrate indole is urgently needed to effectively reduce the level of IS. With the dramatic development of materials science, the biomedical application of advanced materials provides great opportunities for alternative or improved therapeutics. One of the attempts is AST-120, an oral carbon adsorbent that provides a non-dialysis treatment strategy to reduce serum IS level in clinic^11–13^. The mechanism by which AST-120 reduces the level of IS is that it can adsorb the precursor of enterogenous uremic toxins to reduce the levels of toxins in the body. However, it also removes the beneficial nutrients from the intestine, which is mainly ascribed to the non-selectivity caused by the broad pore size of AST-120 and the difficulty in post-modification functionalization^14–16^. Moreover, the pore size of AST-120 is irregular and amorphous, so its adsorption mechanism cannot be directly determined^17^. Therefore, the development of new platforms that surpass current material/strategy and overcome the bottleneck in the field is of great significance.

Covalent organic frameworks (COFs) are an emerging class of porous crystalline materials formed by covalent linkages of small-molecule monomer compounds^18^. COFs possess the advantages of tunable and regular pore size, customizable pore environment, good crystallinity, high stability, facile modification and excellent biocompatibility, which can make up for the disadvantages of AST-120 mentioned above^19^. Thus, COFs have been widely used in adsorption separation^20^, drug delivery^21^, photothermal therapy^22^, and other fields. In particular, it is worth mentioning that the well-defined and adjustable pore structure of COFs is of great help to analyze the mechanism of host-guest interactions, which is also of great significance for the removal of toxins from the body^23^. Hence, COFs provide a potential platform for the selective removal of indole from the intestinal tract, thereby reducing serum IS level. Among all reported COFs, olefin-linked COFs have attracted increasing attention due to their ultrahigh stability, especially water stability. Thus, olefin-linked COFs are promising for surpassing AST-120 in terms of their regular pore size, good crystallinity and functional modification^24,25^. Moreover, the presettable structure and the abundant functional groups (e.g., triazine, pyrazine, pyridine) of olefin-linked COFs provide specific affinity sites, which are conducive to the selective adsorption of indole^26^.

**Fig. 1:**
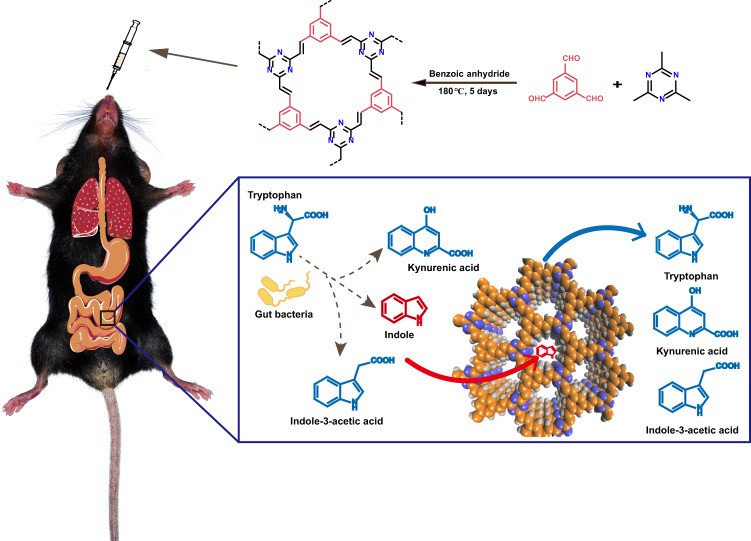
Schematic presentation of this study. Illustration of using COFs as adsorbents to selectively reduce indole intake to delay the progression of chronic kidney disease.

In this study, an ultramicroporous olefin-linked COF prepared in a green and scalable fashion is rationally designed and fabricated as a selective adsorbent of indole in vivo (Fig. 1). Various characterizations and analyses reveal that this olefin-linked COF possesses good gastrointestinal fluid stability, high adsorption efficiency, and excellent biocompatibility. Finally, it is demonstrated that the COF with the optimum adsorption performance and the appropriate pore size exhibits high selectivity toward indole and could significantly attenuate serum IS level in vivo. This study paves a promising avenue for the elimination of IS by non-dialysis strategy and further broadens the in vivo application of COFs.

## Results and discussion

### Design and synthesis of olefin-linked COFs

**Fig. 2:**
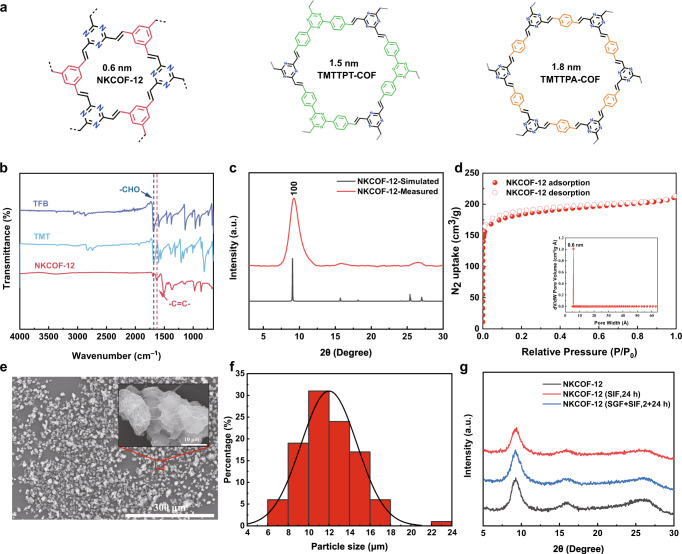
Characterization of NKCOFs. **a** Chemical structures of the COFs. **b** FT-IR spectra of NKCOF-12 and corresponding monomers: TFB (purple), TMT (blue), NKCOF-12 (red). **c** PXRD patterns of NKCOF-12: measured patterns (red), simulated pattern (black). **d** Nitrogen adsorption and desorption isotherms of NKCOF-12. The inset shows the pore size distribution profile of NKCOF-12. **e** SEM image of NKCOF-12 (scale bar = 300 μm, inset: magnified field of view, scale bar = 10 μm). This experiment was independently repeated three times. **f** Histogram of particle size distribution calculated from SEM image. **g** PXRD patterns of NKCOF-12 after treatment with simulated gastric fluid (SGF) and simulated intestinal fluid (SIF).

Olefin-linked COFs have a high degree of structural stability to ensure that they will not degrade in the gastrointestinal tract after oral administration, which is also their main advantage toward in vivo applications^27^. Furthermore, the tunable and regular porous structure of COFs facilitates the elucidation of the adsorption mechanism. Therefore, an ultramicroporous olefin-linked COF (NKCOF-12) for the removal of indole was rationally chosen and prepared from 2,4,6-trimethyl-1,3,5-triazine (TMT) and 1,3,5-triformylbenzene (TFB) followed by melt polymerization^26^. To comprehensively evaluate the confinement effect, we further synthesized two isostructural COFs (TMTTPT-COF and TMTTPA-COF) with different pore sizes for comparison (Supplementary Fig. 1)^26^. The chemical and crystalline structures of olefin-linked COFs (Fig. 2a) were unambiguously characterized by various analytical methods. As shown in Fig. 2b and Supplementary Fig. 2, the existence of new characteristic absorbance bands at 1630 cm^−1^ in the Fourier transform infrared (FT-IR) spectrum revealed the formation of C = C bonds in the olefin-linked COF skeletons. The data in the powder X-ray diffraction (PXRD) spectrum confirmed the successful formation of the targeted COFs with a high degree of crystallinity. The characteristic diffraction peak at 9.3° (Fig. 2c) corresponded to the (100) plane reflections of the hexagonal cells, indicating that NKCOF-12 formed the desired ultramicroporous structure. Similarly, the experimental PXRD patterns of the control materials (TMTTPT-COF and TMTTPA-COF) were also consistent with the simulated PXRD patterns (Supplementary Fig. 3). N_2_ adsorption-desorption experiment was performed to evaluate the porosity and pore size distribution of COFs and AST-120 (Fig. 2d and Supplementary Fig. 4). As shown in Fig. 2d, NKCOF-12 exhibited a typical type I reversible isotherm at 77 K, indicating its ultramicroporous structure with a Brunauer-Emmett-Teller (BET) surface area of 559 m^2^/g. The pore size distribution calculated by nonlinear density functional theory showed that NKCOF-12 had a narrow pore size of 0.6 nm (inset of Fig. 2d). TMTTPT-COF and TMTTPA-COF possessed BET surface areas of 1015 and 1436 m^2^/g and pore sizes of 1.5 nm and 1.8 nm, respectively, which are consistent with the results reported in the literature^26^. In contrast, AST-120 possessed a BET surface area of 1469 m^2^/g and a broad pore size distribution of 0.6–1.8 nm. Scanning electron microscopy (SEM) was performed to investigate the microstructures of COFs with different pore sizes. The results showed that NKCOF-12 and the contrastive COFs possessed similar morphology (Fig. 2e and Supplementary Fig. 5). The particle size distribution of NKCOF-12 was estimated through SEM image, which was between 8–16 μm (Fig. 2f). The contrastive COFs had broad particle size distributions (Supplementary Fig. 6). NKCOF-12 and the contrastive COFs retained superior crystallinity after treatment with gastric and intestinal fluids containing enzymes and aqueous solutions at different pH values (Fig. 2g and Supplementary Figs. 7, 8), displaying the excellent stability required to remove indole from the gastrointestinal tract.

### Capture performance of olefin-linked COFs and AST-120 for indole in the simulated intestinal environment

**Fig. 3:**
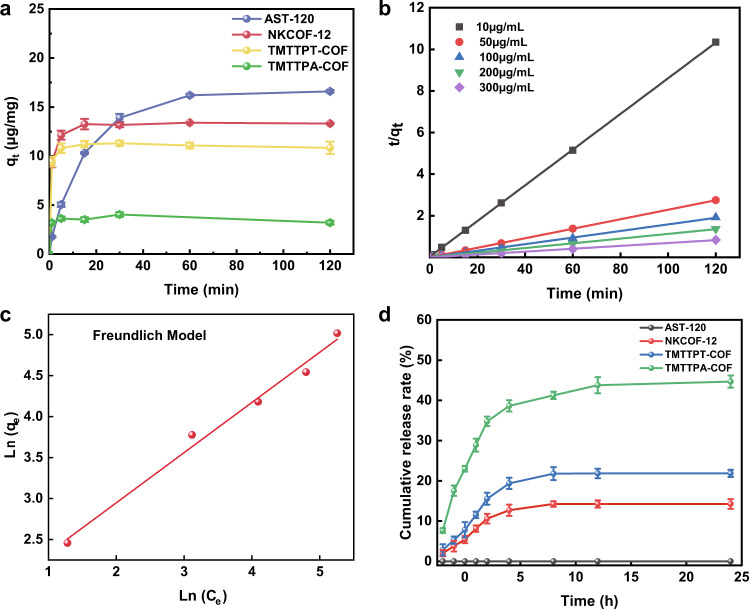
Adsorption experiment of NKCOF-12. **a** Measurement of indole adsorption capacities of olefin-linked COFs (0.5 mg mL^−1^) and AST-120 materials as a function of time. **b** Adsorption kinetic quasi-second-order fitting plots for indole adsorption. **c** Freundlich model fitting plots for indole adsorption isotherms. **d** Cumulative release rates of olefin-linked COFs and AST-120 materials. **a**, **d** Data were presented as the mean ± SD (*n* = 3).

Indole is absorbed into the blood through intestinal epithelial cells and then synthesized into IS by oxidation and sulfation in the liver. Therefore, it is extremely necessary to perform indole adsorption experiments in simulated intestinal fluid to simulate the in vivo application process of COFs. As a result, AST-120 had the highest equilibrium adsorption capacity of 16.51 μg mg^−1^, followed by NKCOF-12 (13.31 μg mg^−1^), TMTTPT-COF (10.84 μg mg^−1^), and TMTTPA-COF (3.200 μg mg^−1^) (Fig. 3a). However, compared with AST-120, the adsorption equilibrium of COFs was quickly reached within 15 min. The superior adsorption kinetics of COFs contributed to adsorbing indole and reducing intestinal intake of indole, thereby decreasing the serum IS level. Two common kinetic models, i.e., quasi-first-order and quasi-second-order kinetic models were used to investigate the adsorption rate of COFs toward indole and the mechanism of the adsorption process (detailed model equations can be seen in the Supplementary Information). Supplementary Fig. 9a and Fig. 3b present the fitting diagrams of the quasi-first-order and quasi-second-order kinetic models for the adsorption of indole on COFs at different initial concentrations (10, 50, 100, 200, and 300 μg mL^−1^). The adsorption kinetic parameters of NKCOF-12 toward indole are listed in Supplementary Table 1. It can be seen that the quasi-second-order model provided a larger value of the correlation coefficient R^2^ (R^2^ > 0.99). The data demonstrated that the adsorption kinetics of NKCOF-12 exhibited better compliance with the quasi-second-order kinetic model, indicating that the rate-limiting step of the capture process of NKCOF-12 toward indole was chemical adsorption. In addition, the kinetic fitting of other olefin-linked COFs was also conducted (Supplementary Table 1).

The adsorption isotherms were explored to evaluate the adsorption capacity of olefin-linked COFs toward indole, in which the initial concentrations ranged from 10 μg mL^−1^ to 300 μg mL^−1^. The Langmuir and Freundlich isotherm models were applicable to monolayer adsorption with a limited number of homogeneous sites and multilayer adsorption of adsorbate on the heterogeneous surface of adsorbents, respectively^28^. In the present study, the obtained adsorption data were modeled with Langmuir and Freundlich isotherm models. The fitting results are shown in Supplementary Fig. 9b and Fig. 3c. The detailed adsorption isotherm parameters of olefin-linked COFs toward indole are listed in Supplementary Table 2. In terms of the higher R^2^ values, the Freundlich model was found to be more suitable for describing the adsorption process of indole on COFs, whereas the Langmuir model mostly fit the adsorption data of AST-120. The results indicated that indole adsorption occurred by a heterogeneous and multilayer mechanism. “n” is the energy heterogeneity factor of the Freundlich model, which is related to the adsorption property^29^. The factor “1/n” indicates the favorability of adsorption^30^. When 1/n is above 1, the adsorption is poor; otherwise, the adsorption reaction is easy^31^. In our study, the 1/n value of NKCOF-12 was 0.6 (Supplementary Table 2), indicating that the adsorption process of indole was easy to carry out.

To clarify how tightly the indole was adsorbed by adsorbents in the intestinal environment, release experiments were performed on materials preloaded with indole using enzyme-free intestinal fluid as the eluent. The results demonstrated that NKCOF-12 possessed the lowest cumulative release rate compared to COFs with larger pore sizes (i.e., TMTTPT-COF and TMTTPA-COF), implying that the appropriate pore size provided tighter adsorption toward indole (Fig. 3d). In addition, PXRD data demonstrated that indole adsorption did not affect the crystallinity degree of the adsorbents (Supplementary Fig. 10).

### Affinity and selectivity of COFs toward indole

**Fig. 4:**
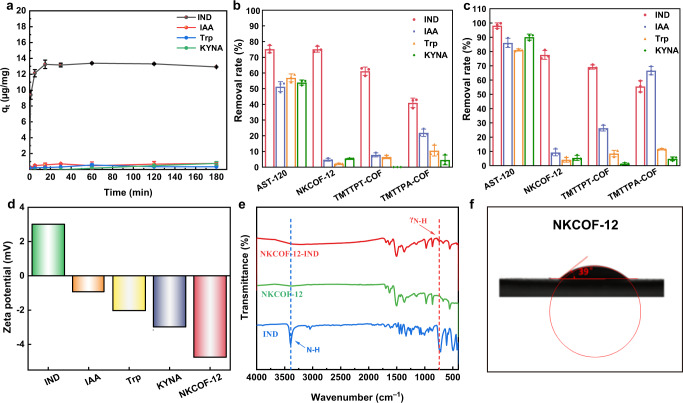
Selective adsorption of NKCOF-12 toward indole. **a** Affinity of NKCOF-12 for indole in a single solution system. **b** Selective removal rate of adsorbents for indole in a multicomponent mixture system within 15 min. **c** Selective removal rate of adsorbents for indole in a multicomponent mixture system within 1 h. **d** Zeta potentials of NKCOF-12 and different compounds. **e** FT-IR spectra of indole (blue), NKCOF-12 (green), and their composite (red). **f** Water contact angle of NKCOF-12. **a**–**c** Data were presented as the mean ± SD (*n* = 3). (IND indole, IAA indole-3-acetic acid, Trp tryptophan, KYNA kynurenic acid).

To avoid the loss of nutrients and drugs in the intestine, the capture process of adsorbents toward indole should be selective. In the present study, indole-3-acetic acid, kynurenic acid and tryptophan, which coexist with indole in the intestine and have similar structures as indole, were selected as the comparison substrates to explore the selective adsorption of COFs toward indole. The experimental results revealed that NKCOF-12 possessed a higher affinity and absorption capacity (13.31 μg mg^−1^) toward indole than the other adsorbents (Fig. 4a and Supplementary Fig. 11). Furthermore, the adsorption selectivity of NKCOF-12 toward indole was investigated by detecting the removal rate of indole from a multicomponent mixture system composed of four compounds (indole, tryptophan, kynurenic acid, and indole-3-acetic acid). As shown in Fig. 4b and Fig. 4c, NKCOF-12 exhibited excellent selective adsorption property for indole, and the adsorption capacity of NKCOF-12 for indole within 15 min was equivalent to that of AST-120, which was conducive to preventing the intestinal uptake of indole in a short time.

**Fig. 5:**
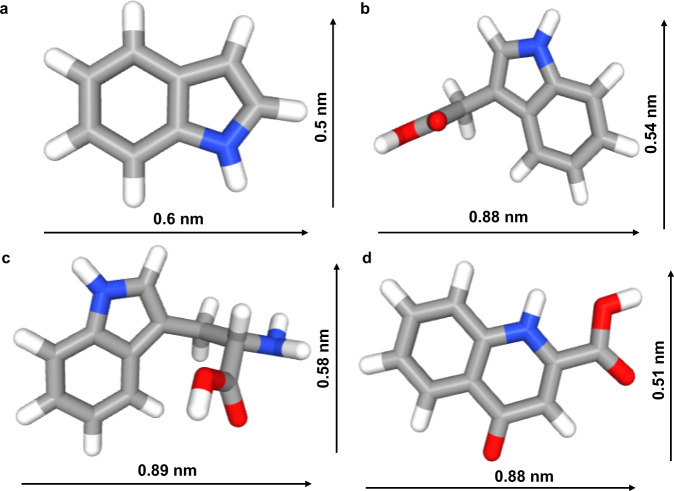
Molecular size of each compound. **a** IND. **b** IAA. **c** Trp. **d** KYNA. (IND indole, IAA indole-3-acetic acid, Trp tryptophan, KYNA kynurenic acid).

According to the literature^32^, the electrostatic force and hydrogen bond are the main factors used to study the host-guest interactions, which provides new insight into the selective adsorption mechanism of NKCOF-12 for indole. The zeta potentials of NKCOF-12, indole, and other substances (indole-3-acetic acid, kynurenic acid, and tryptophan) were determined. Figure 4d illustrated that indole possessed (+) zeta potential, which can be combined with the negatively charged (−) of NKCOF-12 via electrostatic interaction. We hypothesized that the nitrogen-rich triazine ring could bind to the N-H bond of indole to form hydrogen bonds. As shown in Fig. 4e, the NH stretching vibration absorption band at 3400 cm^−1^ of indole became weakened, the γ_N-H_ vibration at 720 cm^−1^ showed a red shift, and a new absorption band at approximately 740 cm^−1^ for NKCOF-12 appeared. All these changes in the infrared absorption spectrum proved the hypothesis of hydrogen bond formation. The measurement results of the water contact angle showed that NKCOF-12 was considered to be a more hydrophilic material with a water contact angle of approximately 39° compared to TMTTPT-COF and TMTTPA-COF (Fig. 4f and Supplementary Fig. 12), and the stronger hydrophilicity of NKCOF-12 contributed to the absorption of indole from the intestinal liquid. In addition, the size selectivity of COFs has been reported to be the basis of the selective interaction between COFs and target compounds^33^. The molecular size (0.5 nm × 0.6 nm) of indole perfectly matched the pore size (0.6 nm) of NKCOF-12 (Fig. 5), indicating stronger guest-host interactions. Given all of that, the high selectivity of NKCOF-12 toward indole resulted from multiple effects, such as size selectivity, electrostatic force, and hydrogen bonding force.

In addition, the competitive adsorption study of COFs toward indole using other nutrients (i.e., glucose, L-phenylalanine, and vitamin B1) contained in intestinal fluid as competitors were also performed. The experimental results showed that NKCOF-12 possessed a higher absorption capacity toward indole than the other adsorbents in a single solution system (Supplementary Fig. 13a–d). Moreover, the competitive adsorption of NKCOF-12 toward indole was investigated by detecting the removal rate of indole from a mixture system composed of three nutrients (glucose, L-phenylalanine, and vitamin B1) and indole. It was found that NKCOF-12 also exhibited excellent selective adsorption property for indole (Supplementary Fig. 13e, f). The above results further confirmed that NKCOF-12 possessed a higher selectivity for indole and can avoid the loss of intestinal nutrients.

### Cytocompatibility and cellular uptake experiment of COFs

**Fig. 6:**
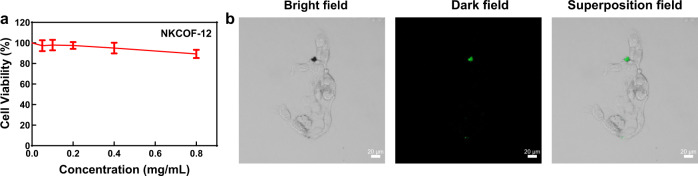
In vitro cytocompatibility and cellular uptake of NKCOF-12. **a** Cell viability of Caco-2 cells after 24 h incubation with different concentrations of NKCOF-12. Data were presented as the mean ± SD (*n* = 6). **b** Confocal images of Caco-2 cells recorded after co-incubation with NKCOF-12 for 24 h. Scale bar = 20 μm. This experiment was independently repeated three times.

The in vitro cytocompatibility of COFs was evaluated using Caco-2 cells by the 3-[4,5-dimethylthial-zol-2-yl]-2,5-diphenyltetrazolium bromide (MTT) method. As shown in Fig. 6a, the MTT assay revealed that the cell viability was over 80% after 24 h incubation with NKCOF-12 when the concentrations ranged from 0.05 to 0.8 mg mL^−1^. It can be considered that NKCOF-12 has no obvious cytotoxicity and possesses good biocompatibility. The cell viability data of the other adsorbents are shown in Supplementary Fig. 14. Cellular uptake was qualitatively assessed using a confocal laser scanning microscope (CLSM), and no fluorescent signal of NKCOF-12 was observed in the cells (Fig. 6b), further suggesting that NKCOF-12 could not be absorbed by cells. The poor cell internalization of NKCOF-12, TMTTPT-COF, and TMTTPA-COF (Supplementary Fig. 15) could mainly be attributed to the repulsive electrostatic interactions between negatively charged COFs and negatively charged cell membranes^34^, as well as the repulsion between hydrophilic NKCOF-12 and lipophilic cell membranes. All the above results fully confirmed that COFs were safe, non-toxic, and could not be absorbed by cells, indicating a great application prospect in the biomedical field. In addition, in vivo biocompatibility and efficacy tests need to be performed when COFs are used for the removal of indole from the intestine. Considering the excellent stability, the highest adsorption efficiency, and the lowest indole release rate of NKCOF-12, it was applied to subsequent animal experiments to further explore the in vivo biocompatibility and effect on serum IS level.

### Animal experiments and detection of serum IS level

To achieve the application of NKCOF-12 on indole removal in vivo and evaluate the indole removal efficacy of NKCOF-12, it is important to assay the in vivo toxicity and efficacy of NKCOF-12 and detect the content of serum IS. In the present study, the in vivo toxicity of NKCOF-12 was first evaluated in C57BL/6 J mice. The mice were randomly divided into normal group and NKCOF-12 group. We evaluated the effect of NKCOF-12 on the body weight and organ weight index of normal mice. The results (Supplementary Fig. 16) indicated that the body weight and organ weight index of mice in the NKCOF-12 group were not significantly abnormal compared with the normal group. Moreover, the effect of NKCOF-12 on major organs (heart, liver, spleen, lung, kidney, stomach, and intestine) was further evaluated by hematoxylin and eosin (H&E) staining. H&E staining results (Supplementary Fig. 17) showed no significant tissue damage or pathological change in any of the major organs after oral administration of NKCOF-12 for 8 weeks. Finally, serum biochemistry was performed, and the results showed no obvious change in the liver (alanine aminotransferase (ALT) and aspartate aminotransferase (AST)) or kidney (blood urea nitrogen (BUN) and creatinine (Cr)) function after 8 weeks (Supplementary Fig. 18). All the above toxicity assays confirmed the high biosafety of NKCOF-12, indicating that COFs have a great potential for in vivo application. Inspired by the high biosafety of NKCOF-12 in vivo, we further investigated the efficacy of NKCOF-12 in CKD mice and explored its effect on the uremic toxin IS in vivo. Thirty-two mice were randomly divided into 4 groups containing the normal, model, NKCOF-12 and AST-120 groups. The symptoms of adenine-induced chronic kidney injury in mice are basically the same as the clinical features of CKD in humans. According to the methods reported in the literature^35^, the mouse CKD model was established by adenine diet induction except for the normal group (Supplementary Fig. 19). Cr and BUN are the most important test indexes reflecting renal function. To investigate whether the model was successfully established, H&E staining of kidney tissue from mice in the normal and model groups was carried out, and the serum levels of Cr and BUN in the two groups were detected. Elevated serum Cr and BUN levels (Supplementary Fig. 20) and the abnormal pathology of H&E staining of the kidney (Supplementary Fig. 21) indicated that the CKD model was successfully established.

**Fig. 7:**
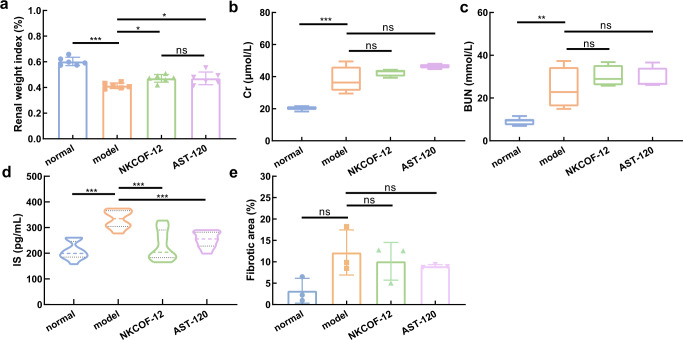
Therapeutic effect of NKCOF-12 on CKD mice. **a** Kidney weight index of mice in different groups (*n* = 6). **b** Serum Cr level of mice in different groups (*n* = 6). **c** Serum BUN levels of mice in different groups (*n* = 6). **d** Serum IS level of mice after drug intervention for 8 weeks (*n* = 6). **e** Collagen fiber area percentage of kidney measured by Masson staining (*n* = 3). **a** Data were represented as the mean ± SD. **b**, **c** Data presented as box-and-whisker plots, whiskers represent minima and maxima. * *P* < 0.05, **P < 0.01, ***P < 0.001, ns: no significance. Statistics were calculated by one-way ANOVA followed by Tukey’s post-test. Exact *P* values are given in the Supporting Information file.

After successfully modeling, the CKD mice in the NKCOF-12 and AST-120 groups received oral gavages of NKCOF-12 and AST-120, respectively. The effect of drug intervention on renal pathology as well as serum IS level in CKD mice were assessed. Body weight and kidney weight index in the model group were significantly decreased compared with those in the normal group (*P* < 0.001) (Supplementary Fig. 22 and Fig. 7a). The kidney weight index in NKCOF-12 and AST-120 groups were higher than that in the model group (*P* < 0.05), whereas there was no significant difference between the NKCOF-12 and AST-120 groups (*P* > 0.05). These results proved that both NKCOF-12 and AST-120 could delay renal damage to some extent. Meanwhile, the serum Cr and BUN levels of the NKCOF-12 and AST-120 groups were not significantly different from those of the model group (Fig. 7b, c, P > 0.05), suggesting that NKCOF-12 and AST-120 had no obvious effect on renal function indexes of CKD mice. In addition, the serum IS levels in different groups after oral administration of NKCOF-12 and AST-120 for 8 weeks were directly quantified by an enzyme-linked immunoassay kit. Figure 7d clearly shows that NKCOF-12 and AST-120 significantly reduced the serum IS level compared to the model group (*P* < 0.001). The results fully demonstrated that NKCOF-12 could effectively remove indole, thereby reducing the serum IS level. Moreover, NKCOF-12 had the same efficacy as AST-120. Therefore, it is feasible to use NKCOF-12 as an alternative to AST-120 to reduce the serum IS level of CKD mice.

**Fig. 8:**
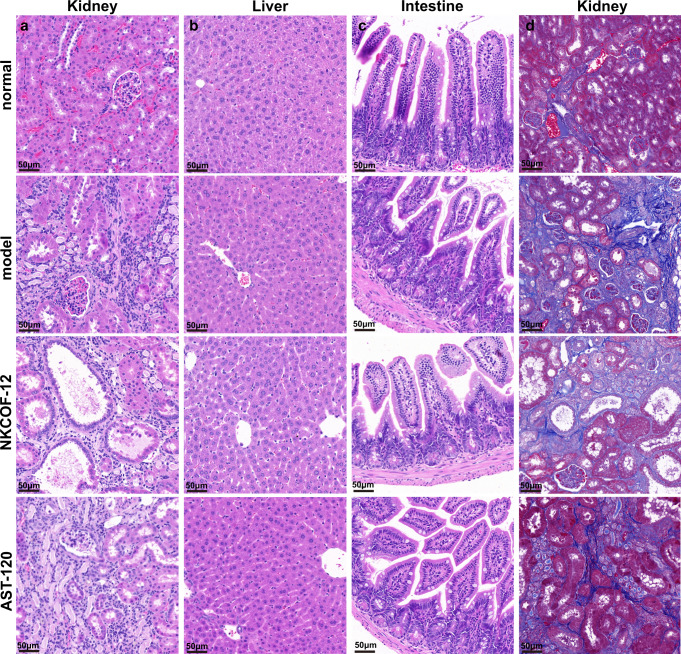
Histopathological staining. **a** Micrographs of H&E-stained kidney slices from mice of different groups. **b** Micrographs of H&E-stained liver slices from mice of different groups. **c** Micrographs of H&E-stained intestine slices from mice of different groups. **d** Micrographs of Masson trichrome-stained kidney slices from mice of different groups. Scale bar = 50 μm. Each experiment was independently repeated three times.

Furthermore, the micrographs of H&E staining of renal tissue showed that there was obvious necrosis, inflammatory cell infiltration and fibrosis in the renal tubules of the model group, while NKCOF-12 and AST-120 treatment did not reverse the tissue damage (Fig. 8a). Masson staining, in which collagen fibers are stained blue and myofibril cytoplasm is dyed red, has become a means of detecting tissue fibrosis^36^. As displayed in Fig. 8d, more collagen fiber deposition occurred in the kidney of the model group compared with the normal group, and the area of fibrosis decreased after NKCOF-12 and AST-120 intervention, but there was no significant difference (*P* > 0.05) (Fig. 7e). The experimental data further confirmed that NKCOF-12 can effectively slow the progression, although it cannot reverse the renal injury. From the H&E staining micrographs of liver and intestine tissues, we can see that there was no obvious liver and intestine damage in the NKCOF-12 group (Fig. 8b, c). And, we found that there was no significant difference in liver weight index and liver function indexes between the two groups (Supplementary Figs. 23, 24). These results further confirmed that NKCOF-12 had no side effects on the mice and exerted excellent in vivo biocompatibility, which could be attributed to the fact that NKCOF-12 cannot be absorbed by the intestinal tract and will not produce hepatotoxicity through blood circulation.

To evaluate the selectivity of NKCOF-12 in a more comprehensive manner, bowel contents of mice with or without NKCOF-12/AST-120 treatment were analyzed based on animal experiments. Tryptophan and its metabolites (i.e., indole, indole-3-acetic acid, kynurenic acid, tryptamine, indole-3-carboxaldehyde, and indole-3-propionic acid) as well as several nutrients (i.e., L-phenylalanine and vitamin B1) in cecal contents of mice of different groups were quantified by ultra-high-performance liquid chromatography coupled with triple-quadrupole linear ion-trap tandem mass spectrometry (UHPLC-QTRAP-MS/MS). According to the literature, the concentration of indole in bowel contents is affected by the diet and living environment of mice, which range from 4.76 µg g^−1^ to 9.39 µg g^−1^ ^37^. In our study, the concentration of indole in normal mice was 5.12 ± 1.13 µg g^−1^, which was consistent with the results reported in the literature. However, the concentration of indole in the model group was 7.19 ± 1.75 µg g^−1^, slightly higher than that of the normal group, which may be contributed to the gut microbiome dysbiosis in CKD disease, thus causing the formation of potentially toxic metabolites, such as indole^7^. After treatment with NKCOF-12 and AST-120, the concentration of indole in intestinal contents was 3.62 ± 0.36 µg g^−1^ and 3.92 ± 0.74 µg g^−1^, respectively, significantly lower than that of the model group (*P* < 0.01) (Supplementary Fig. 25). The results fully demonstrated that NKCOF-12 could effectively remove indole, thereby reducing the serum IS level. Furthermore, NKCOF-12 could effectively remove indole from the intestine without causing the loss of other substances compared with AST-120, thus avoiding the loss of intestinal nutrients and drugs. In combination with the previous selective and competitive adsorption results of COFs and AST-120, it was further confirmed that NKCOF-12 possessed a higher selectivity toward indole than AST-120.

In conclusion, an ultramicroporous olefin-linked COF (NKCOF-12) with good biocompatibility and excellent adsorption selectivity for indole was successfully prepared via the melt polymerization synthesis method. Various experimental results and analyses demonstrate that NKCOF-12 exerts the following advantages: (1) the synthesis method without solvent consumption conforms to the concept of green synthesis; (2) it exhibits good stability under the simulated gastrointestinal pH conditions; (3) it possesses an adjustable pore size and displays excellent selective adsorption property for indole; (4) it has a good cytocompatibility, thereby reducing the risk of toxicity; (5) NKCOF-12 has no side effects owing to its good in vivo biocompatibility, which effectively reduced the serum IS level. In addition, NKCOF-12 exhibits a higher selectivity adsorption property toward indole compared with the commercially available adsorbent AST-120 that has been used clinically, which renders it highly attractive and promising for uremic toxin removal. Hence, the present study opens up new ideas for the elimination of IS by a non-dialysis strategy based on COFs and expands the in vivo applications of COFs. Meanwhile, this study provides new research ideas for delaying the progression of aristolochic acid-induced renal injury, the developed non-dialysis strategy will have a great application prospect in the treatment of herbal medicine-induced nephropathy.

## Methods

### Green synthesis of olefin-linked COFs with different pore sizes

In a typical synthesis, TFB (64.8 mg, 0.4 mmol), TMT (49.2 mg, 0.4 mmol), and benzoic anhydride (271.5 mg, 1.2 mmol) were weighed into a Pyrex tube. The tube was degassed through three freeze-pump-thaw cycles and sealed under vacuum. Then, the tube was transferred into an oven where the sample was heated at 180 °C for 5 days. After the reaction was completed, the yellow solid was collected, washed with DMF and methanol, and dried at 100 °C under vacuum for 12 h to afford NKCOF-12. The synthesis method of the control COFs is similar to that of NKCOF-12. The detailed methods can be seen in the Supplementary information.

#### Indole adsorption experiment

The adsorption experiment of indole by COFs was conducted by ultraviolet-visible spectrometry. 10 mg of NKCOF-12 or COFs with different pore sizes was added to 20 mL indole solution (10 µg mL^−1^). The mixture was incubated at 37 °C for 180 min, and the concentration of the residual indole in the supernatant was measured at 270 nm. The amount of indole captured by COFs was calculated using the following Eq. (1). All experiments were performed in triplicate, and the average values were used for data analysis.

1 $${q}_{t}=\frac{\left({C}_{0-}{C}_{t}\right)\times V}{m}$$

Where *q*_*t*_ (µg mg^−1^) is the mass of indole that was adsorbed per unit mass of COFs at time t, *C*_*t*_ (µg mL^−1^) is the concentration of residual indole in the supernatant, *C*_*0*_ (µg mL^−1^) is the initial concentration of indole, *V* (mL) is the volume of the adsorption system, and *m* (mg) is the mass of COFs.

The detailed research procedures of the release experiment of indole as well as the adsorption kinetics and adsorption isotherms of COFs toward indole are described in the Supplementary information.

#### Selectivity absorption experiment of COFs

Indole and its structural analogs (tryptophan, kynurenic acid, and indole-3-acetic acid) were dissolved in the simulated intestinal fluid and mixed with different COFs to give the final concentrations of 10 µg mL^−1^ for each compound and 0.5 mg mL^−1^ for COFs. The mixture was incubated in an incubator shaker at 37 °C. The concentrations of the residual indole and its analogs in the supernatant were detected using an Agilent 1260 Infinity HPLC (Agilent Technologies, Santa Clara, CA, USA) equipped with a Diamonsil C_18_ column (200 mm × 4.6 mm, 5 µm). The mobile phase (flow rate 0.6 mL min^−1^) consisted of acetate buffer at pH 5.4 and acetonitrile. The detection wavelengths were set at 280 nm for indole, tryptophan, and indole-3-acetic acid and 332 nm for kynurenic acid using an ultraviolet detector. The competitive adsorption study of indole by COFs in the presence of other nutrients (glucose, L-phenylalanine, and vitamin B1) was performed by following the same operation procedure as above. The residual indole and nutrients were detected on the same HPLC system. Chromatographic separation was achieved on the same C_18_ column using a mobile phase consisting of methanol-0.1% formic acid in water with gradient elution (flow rate 1.0 mL min^−1^). The detection wavelengths were set at 280 nm for indole, and 258 nm for vitamin B1 and L-phenylalanine using a diode-array detector. The concentration of glucose was measured using a glucose test kit (O-toluidine, Beyotime Biotechnology Co. Ltd., Shanghai, China, Lot. No 081022221026). The reference materials (purity ≥98%) used were purchased from Sigma-Aldrich Corp. (St. Louis, MO, USA) and Shanghai Yuanye Bio-Technology Co., Ltd. (Shanghai, China).

#### Cytocompatibility and cellular uptake experiment of COFs

The cytocompatibility of COFs was assayed in Caco-2 cells. Caco-2 cell line was purchased from Procell Life Science & Technology Co., Ltd. (Wuhan, China). Caco-2 cells were seeded into 96-well plates at a density of 5 × 10^3^ cells per well and then cultured in minimum Eagle’s medium (MEM; Procell) containing 20% fetal bovine serum at 37 °C with 5% CO_2_ for 24 h. The medium was then changed to fresh medium containing four different materials, namely, NKCOF-12, TMTTPT-COF, TMTTPA-COF and milled AST-120, with the final action concentration of 0.05 to 0.8 mg mL^−1^. After incubation for 48 h, the medium was discarded and replaced with 200 μL of fresh medium containing 10 μL MTT (5 mg mL^−1^). The cells continued to be incubated at 37 °C with 5% CO_2_ for 4 h. After discarding the medium, 150 μL of dimethyl sulfoxide (DMSO; Sigma-Aldrich Corp.) was added to dissolve the formazan crystals, and the absorbance at 490 nm was recorded on a microplate reader. The experiment was repeated six times.

The cellular uptake of COFs was evaluated using a CLSM imaging system. Caco-2 cells at a density of 5 × 10^3^ were seeded in CLSM special dishes and cultured for 48 h. After replacement of the fresh medium, the COFs were added to each dish to give a final action concentration of 20 µg mL^−1^. Then, the cells were washed 3 times with PBS after co-cultivation for 24 h. Caco-2 cells were fixed with 4% paraformaldehyde for 20 min before recording with a confocal imaging system.

#### Animal experiments

The in vivo biocompatibility and efficacy of NKCOF-12 were evaluated using male C57BL/6 J mice (6 weeks old). The mice were purchased from the Beijing Military Medical Science Academy of the PLA (Beijing, China). They were adaptively housed in the SPF facility for one week under a 12 h light/dark cycle, with free access to water and Sterilized AIN93G pellets (Beijing HFK Biotechnology Co., Ltd., China). Ambient temperature is about 25 °C and the relative humidity is 55–60%. All animal experiments were approved by the Care and Use Committee of the Animal Experiment Center of Tianjin University of Traditional Chinese Medicine. The in vivo biocompatibility was examined following oral administration of NKCOF-12 at a dose of 40 mg kg^−1^ d^−1^ for 8 weeks. The mice were euthanized, and the major organs (heart, liver, spleen, lung, kidney, stomach, and intestine) were collected, embedded in paraffin, and sectioned. Then, histological analysis was performed with H&E staining. Moreover, blood samples collected from the eyeball were used for biochemical examination. The efficacy of NKCOF-12 on CKD mice was evaluated. The mouse model of CKD was established by adenine (0.2%) diet induction for 6 weeks. The renal function indexes (Cr and BUN) and H&E staining of kidney tissue were used to evaluate whether the modeling was successful. CKD mice in the NKCOF-12 and AST-120 groups were administrated with NKCOF-12 and AST-120 at a dose of 40 mg kg^−1^ d^−1^ for 8 weeks, respectively, while the mice in the normal and model groups were perfused with the same volume of vehicle solution (olive oil). At the end of the experiment, animals in different groups were euthanized, and liver, kidney, and intestinal tissues were collected for H&E staining and Masson staining. The serum IS level was measured by enzyme-linked immunosorbent assay kit (Lot. YT004274-96T) that was purchased from Tianjin EterLife Science Research and Development Co., Ltd. (Tianjin, China). The bowel contents with or without NKCOF-12/AST-120 treatment were analyzed by UHPLC-QTRAP-MS/MS. The detailed operation procedures can be found in the Supplementary information.

#### Statistical analysis

Data are presented as the mean ± standard deviation (SD). Data from experiments were analyzed with Origin 2019. Statistical analysis was performed with statistical software (GraphPad Prism v8.0 and IBM SPSS Statistics v21.0). Two-tailed one-way analysis of variance (ANOVA) followed by Tukey’s post hoc test was used for comparisons among multiple groups. Independent sample *t*-test (two-sided test) was used for comparison between the two groups (*P < 0.05; **P < 0.01; ***P < 0.001; ns: no significance).

### Reporting summary

Further information on research design is available in the Nature Portfolio Reporting Summary linked to this article.

## Supplementary information

Supplementary Information

Reporting Summary

## Data Availability

All data supporting the findings of this study are available within the article, as well as the Supplementary Information file, or available from the authors on request. Source data are provided in this paper.

## Source data

Source Data
