# Supplementary material for: Efficient selective removal of uremic toxin precursor by olefin-linked covalent organic frameworks for nephropathy treatment
Source: Nat Commun. 2023 May 16;14:2805. doi: 10.1038/s41467-023-38427-3 (PMC10188479; doi:10.1038/s41467-023-38427-3)
Supplement: Supplementary file 1 — Supplementary Information [file 41467_2023_38427_MOESM1_ESM.pdf]

Supplementary Information

Efficient selective removal of uremic toxin precursor by olefin-linked covalent organic frameworks for nephropathy treatment

Jinxia Wei[1,#], Rui Li[1,#], Penghui Zhang[2,#], Haiqun Jin[2], Zhenjie Zhang[3], Yubo Li[1] * and Yao Chen[2] *

[1] School of Chinese Materia Medica, Tianjin University of Traditional Chinese Medicine, Tianjin 301617, China

[2] State Key Laboratory of Medicinal Chemical Biology, College of Pharmacy, Nankai University, Tianjin 300071, China

[3] College of Chemistry, Nankai University, Tianjin 300071, China

[#]These authors contributed equally: Jinxia Wei, Rui Li, Penghui Zhang.

*Correspondence:

E-mail address: yaowufenxi001@sina.com; chenyao@nankai.edu.cn

Table of Contents

1. Supplementary Methods 3

1.1 Chemicals and Characterization 3

1.2 Synthesis of TMTTPT-COF 3

1.3 Synthesis of TMTTPA-COF 3

1.4 Release experiment of indole 4

1.5 Adsorption kinetics and adsorption isotherms of COFs toward indole 4

1.6 Determination of indoxyl sulfate (IS) in mice serum 5

1.7 Mass spectrometric analysis of bowel contents of mice.................6

2. Supplementary Tables 1-2 7

3. Supplementary Figures 1-22 8

4. Supplementary References...20

1. Supplementary Methods

1.1 Chemicals and Characterization

Except for a special description, all materials used in the experiments were purchased from commercial sources and used without further purification.

^{1}H NMR spectra were recorded on Bruker AV400 instruments (400 MHz). Powder X-ray diffraction (PXRD) measurements were recorded on a D/Max-2500 X-ray diffractometer by depositing powder on the glass substrate, 2θ ranged from 1.5° to 30° with 0.02° increment. The adsorption measurements of gas were performed using a Micromeritics ASAP 2460. Before analysis, the samples were degassed under vacuum at 120 °C for 12 h. Fourier transform infrared (FT-IR) spectra were recorded on Nicolet iS50 FT-IR Spectrometer. Scanning electron microscopy (SEM) images were observed by Hitachi SU3500 scanning electron microscope.

1.2 Synthesis of TMTTPT-COF

In a typical synthesis[1], TPT (157.4 mg, 0.4 mmol), TMT (49.2 mg, 0.4 mmol), and benzoic anhydride (271.5 mg, 1.2 mmol) were weighed into a Pyrex tube. The tube was degassed through three freeze-pump-thaw cycles and sealed under vacuum. Then the tube was transferred into an oven where the sample was heated at 180 °C for 5 days. After the reaction was completed, the yellow solid was collected, washed with DMF and methanol, and dried at 100 °C under vacuum for 12 h to afford TMTTPT-COF.

1.3 Synthesis of TMTTPA-COF

In a typical synthesis[1], TPA (80.4 mg, 0.4 mmol), TMT (49.2 mg, 0.4 mmol), and benzoic anhydride (271.5 mg, 1.2 mmol) were weighed into a Pyrex tube. The tube was degassed through three freeze-pump-thaw cycles and sealed under vacuum. Then

the tube was transferred into an oven where the sample was heated at 180 °C for 5 days. After the reaction was completed, the yellow solid was collected, washed with DMF and methanol, and dried at 100 °C under vacuum for 12 h to afford TMTTPA-COF.

1.4 Release experiment of indole

The COFs were activated to investigate the release profile. Firstly, the indole was adsorbed by 10 mg COFs in the simulated intestinal fluid (SIF), and the experimental conditions were consistent with those of the adsorption experiment. After absorbing for 180 min, the sample was centrifuged and the supernatant was removed. Then, the indole@COFs composite was washed with SIF to remove indole from the material surface. 1 mL SIF was added to investigate the release of indole from the indole@COFs composite. The SIF was replaced with new intestinal fluid at intervals. Finally, the release amount of indole from the indole@COFs composite was quantified by the Ultraviolet-visible spectrophotometer.

1.5 Adsorption kinetics and adsorption isotherms of COFs toward indole

10 mg of activated COFs was added into 20 mL indole solution with a series of different concentrations (10, 50, 100, 200, and 300 μg mL^{-1}). Two common kinetic models (i.e., quasi-first-order and quasi-second-order kinetic models) were used to investigate the adsorption rate of COFs toward indole. The quasi-first-order kinetic model can be expressed by the following linear equation (1):

$$ln(q_e - q_t) = lnq_{e,\ cal,1} - k_1 t \cdots\cdots(1)$$

The quasi-second-order kinetic model can be represented by the following linear equation (2):

$$\frac{t}{q_t} = \frac{1}{k_2 q_{e,cal,2}^2} + \frac{t}{q_{e,cal,2}} \cdots\cdots(2)$$

Where q_e and q_t are the indole adsorption capacities (mg g^{-1}) at equilibrium and time t (min), respectively; $q_{e,cal,1}$ and $q_{e,cal,2}$ are the equilibrium adsorption capacities (mg g^{-1})

that are calculated according to the quasi-first-order and quasi-second-order models, respectively; k_1 (min^{-1}) and k_2 (g mg^{-1} min^{-1}) are the adsorption rate constants of the quasi-first-order equation and quasi-second-order equation, respectively. $h = k_2q^2_{e,cal,2}$ is the initial adsorption rate (mg g^{-1} min^{-1}) that is calculated using the quasi-second-order model.

The adsorption behavior was simulated using the classical Langmuir and Freundlich isotherm models. The two isotherm models represent different adsorption processes. The Langmuir isotherm model assumes the formation of a monolayer of adsorbate on the surface of a homogeneous adsorbent during the adsorption process, while the Freundlich isotherm model is an empirical equation that takes into account the inhomogeneity of adsorption on the adsorbent surface in multiple layers. The two models are defined as the following equations (3)-(4):

Langmuir isotherm model:

$$\frac{C_e}{q_e} = \frac{1}{K_e q_m} + \frac{C_e}{q_m} \cdots\cdots (3)$$

Freundlich isotherm model:

$$ln\, q_e = ln\, K_F + \left(\frac{1}{n}\right) ln\, C_e \cdots\cdots (4)$$

Where C_e (mg L^{-1}) is equilibrium concentration of indole; q_m (mg g^{-1}) is the maximum theoretical adsorption capacity; K_e (L/mg) and K_F (mg g^{-1}) $(L/mg)^{1/n}$ are the Langmuir and Freundlich equilibrium adsorption constants, respectively; "*1/n*" is the heterogeneity factor.

1.6 Determination of indoxyl sulfate (IS) in mice serum

Serum IS levels were detected by a double antibody sandwich enzyme-linked immunosorbent assay (ELISA). The sample, standard, and HRP-labeled detection antibody were added to microwells pre-coated with IS antibody in sequence, incubated and washed thoroughly. The color was developed with the substrate 3, 3', 5, 5'-tetramethylbenzidine, which was converted into blue under the catalysis of peroxidase and finally into yellow under the action of acid. The shade of color is

positively correlated with the concentration of IS in the sample. The absorbance (OD value) was measured with a microplate reader at a wavelength of 450 nm, which was used to calculate the content of IS in the sample.

1.7 Mass spectrometric analysis of bowel contents of mice

The selectivity of NKCOF-12 has been checked in a more comprehensive manner by mass spectrometric analysis. Tryptophan and its metabolites (i.e., indole, indole-3-acetic acid, kynurenic acid, tryptamine, indole-3-carboxaldehyde, and indole-3-propionic acid) as well as several nutrients (i.e., L-phenylalanine and vitamin B1) in cecal contents of mice with or without NKCOF-12/AST-120 treatment were quantified by ultra-high-performance liquid chromatography coupled with triple-quadrupole linear ion-trap tandem mass spectrometry (UHPLC-QTRAP-MS/MS). The standards of tryptophan and its metabolites (purity ≥98%) were purchased from Sigma-Aldrich Corp. (St. Louis, MO, USA). The standards of L-phenylalanine and vitamin B1 (purity ≥98%) were obtained from Shanghai Yuanye Bio-Technology Co., Ltd. (Shanghai, China).

Sample preparation: The sample was placed in a mortar and underwent cryogenic grinding in liquid nitrogen. 30 mg of powdered sample was weighed and mixed with 500 μL acetonitrile/methanol/water (2:2:1, v/v) mixed solvent. Then, 10 μL internal standard (2 μg mL^{-1}) was added into the mixture. After vortexing for 10 min, the mixture was subjected to ultrasonic treatment for twice in turn, each time 30 min. The sample was placed at -20 °C for 1 h to precipitate protein. After centrifugation at 4 °C for 20 min at 14000 ×g, the supernatant was transferred to a microcentrifuge tube and freeze dried. The dried samples were redissolved in mobile phase and centrifuged at 4 °C for 20 min. The supernatant was then analyzed using UHPLC-QTRAP-MS/MS.

UHPLC and MS conditions: Chromatographic analysis was performed on an Agilent 1290 Infinity UHPLC system (Agilent Technologies, Santa Clara, CA, USA). A Waters ACQUITY UPLC CSH C_{18} column (2.1 mm × 100 mm, 1.7 μm, Waters Corp., USA) with an oven temperature at 40 °C was used for chromatographic

isolation. The mobile phases consisted of water (containing 20 mM ammonium formate and 0.1% formic acid) and methanol (containing 0.1% formic acid) with a flow rate of 0.4 mL min^{-1}. A 5 μL aliquot of sample solution was injected into the UHPLC-QTRAP-MS/MS system for analysis. Quality control samples are inserted into the sample queue every certain number of experimental samples to detect and evaluate the stability and repeatability of the system. A triple quadrupole-linear ion trap mass spectrometer (QUAD-5500; AB SCIEX, Framingham, MA, USA) was used to perform data acquisition in both positive and negative ion modes. Operating parameters were set as follows: source temperature, 450 °C; ion source gas (gas 1), 45 psi; ion source gas (gas 2), 45 psi; curtain gas, 40 psi; ion spray voltage floating, 4500 V (positive mode)/-4500 V (negative mode). MRM mode was used to detect the quantitative ion pairs. The peak annotation and quantification were performed using Multiquant 3.0.2 (AB SCIEX, Framingham, MA, USA).

2. Supplementary Tables 1-2

Supplementary Table 1 Quasi-first-order and quasi-second-order kinetic models and their statistical parameters.

Adsorbents	$q_{e,exp}$ (mg g^{-1})	Quasi-first-order model			Quasi-second-order model			
		$q_{e,cal,1}$ (mg g^{-1})	K_1 (min^{-1})	R^2	$q_{e,cal,2}$ (mg g^{-1})	K_2 (g mg^{-1} min^{-1})	h (mg g^{-1} min^{-1})	R^2
NKCOF-12	13.31	1.556	0.0285	0.4572	11.65	0.2667	36.20	0.9999
TMTTPT-COF	10.84	6.130	0.05089	0.8447	11.89	0.0354	5.010	0.9997
TMTTPA-COF	3.200	2.287	0.1910	0.8892	4.007	0.2837	4.556	0.9935
AST-120	16.51	10.09	0.01613	0.7594	17.59	0.006196	1.917	0.9985

Supplementary Table 2 Relevant parameters for Langmuir and Freundlich isotherm equations.

Adsorbents	Langmuir isotherm equation			Freundlich isotherm equation		
	q_m (mg g^{-1})	K_e (L/mg)	R^2	K_F (mg g^{-1}) $(L/mg)^{1/n}$	1/n	R^2
NKCOF-12	190.8397	431.5589	0.8365	5.5991	0.6126	0.9897
TMTTPT-COF	384.6153	2766.2211	0.6442	17.6973	0.5485	0.9784
TMTTPA-COF	315.4574	1115.9128	0.4269	0.4242	1.0498	0.9798
AST-120	293.2551	21052.0554	0.9903	72.8258	0.3201	0.9760

3. Supplementary Figures 1-19

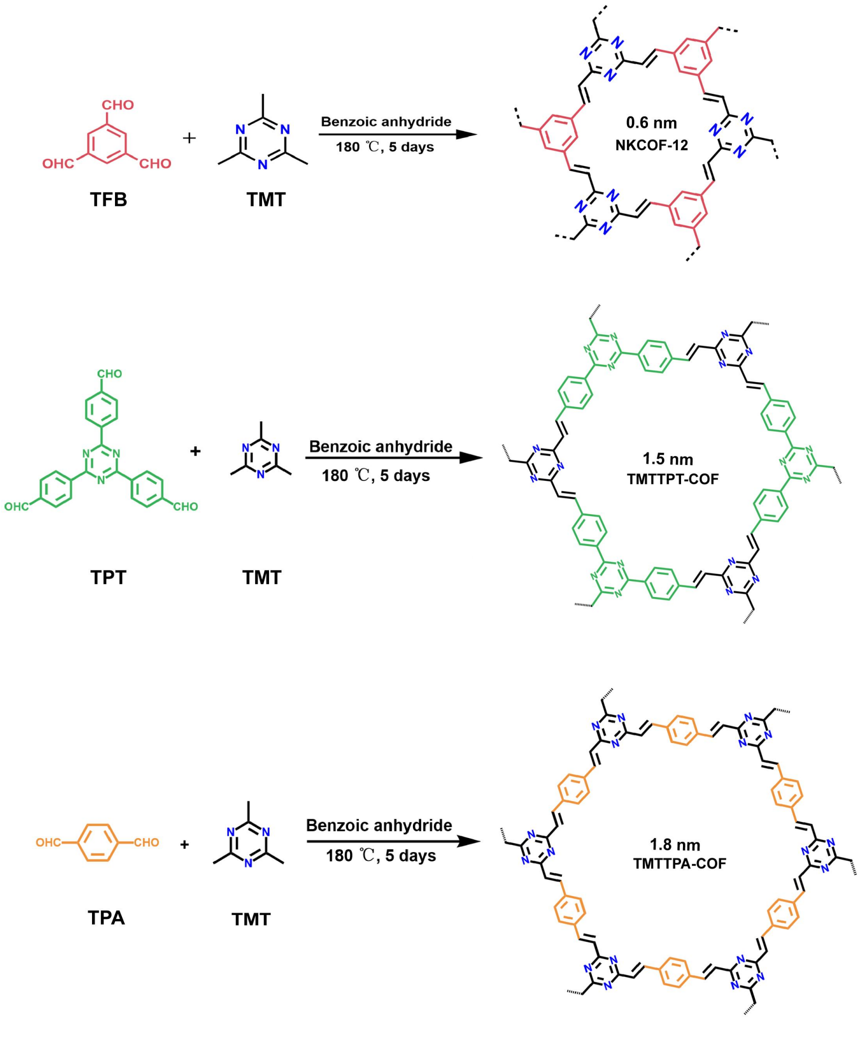

Supplementary Fig. 1 Schematic illustration of the synthesis of NKCOF-12, TMTTPT-COF, and TMTTPA-COF.

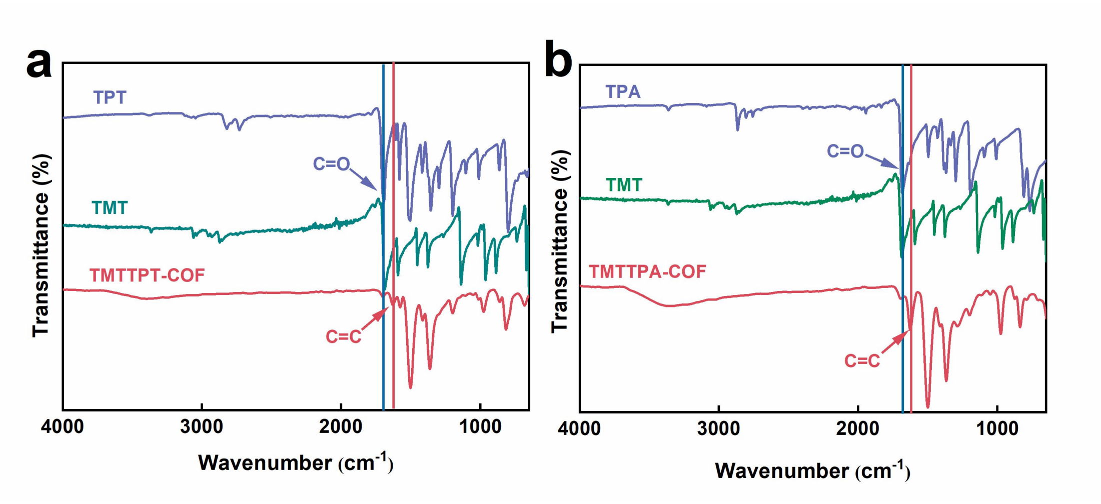

Supplementary Fig. 2 FT-IR spectra of TMTTPT-COF (**a**) and TMTTPA-COF (**b**).

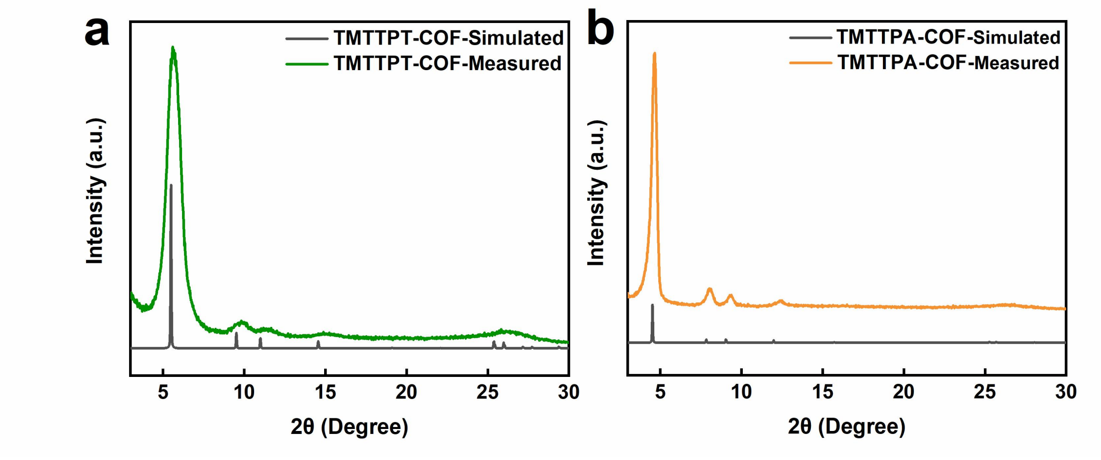

Supplementary Fig. 3 PXRD patterns of TMTTPT-COF (**a**) and TMTTPA-COF (**b**).

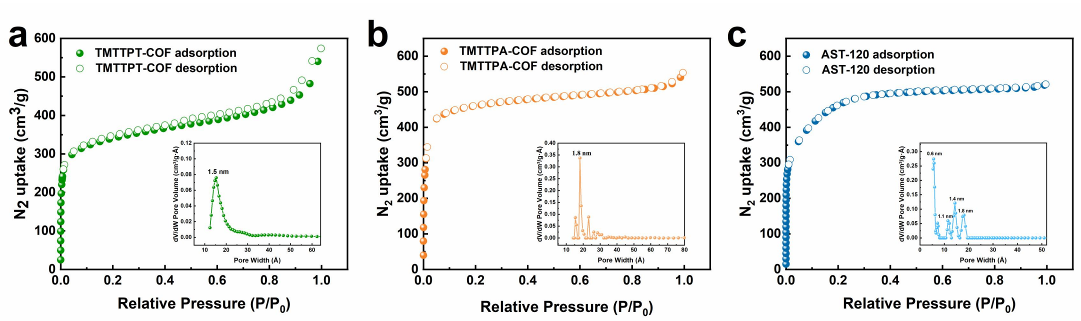

Supplementary Fig. 4 Nitrogen adsorption and desorption isotherms and pore size distribution of TMTTPT-COF (**a**) TMTTPA-COF (**b**) and AST-120 (**c**).

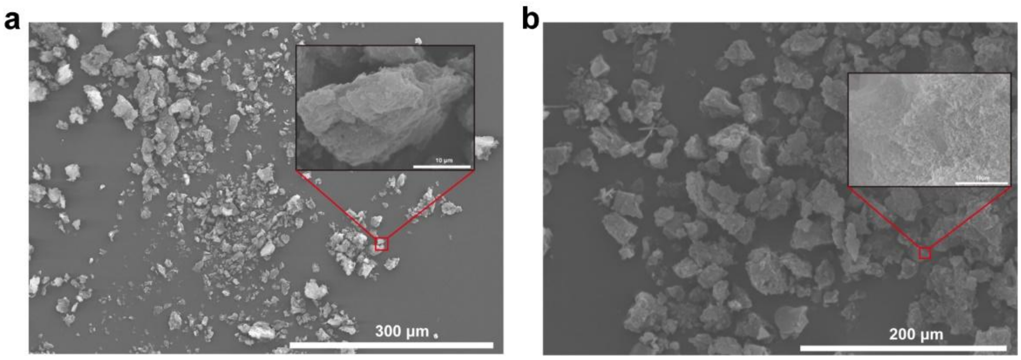

Supplementary Fig. 5 SEM images of TMTTPT-COF (**a**, scale bar = 300 µm, inset: magnified field of vision, scale bar = 10 µm) and TMTTPA-COF (**b**, scale bar = 200 µm, inset: magnified field of vision, scale bar = 10 µm). Each experiment was independently repeated three times.

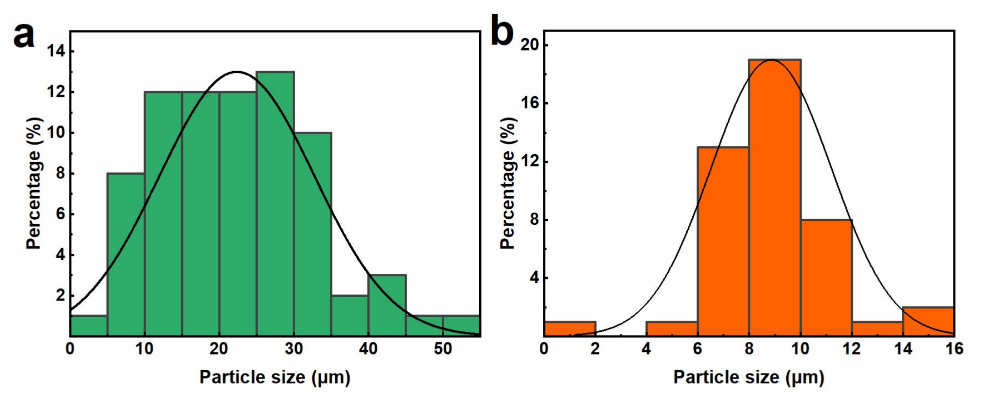

Supplementary Fig. 6 Size distribution histograms of TMTTPT-COF (**a**) and TMTTPA-COF (**b**).

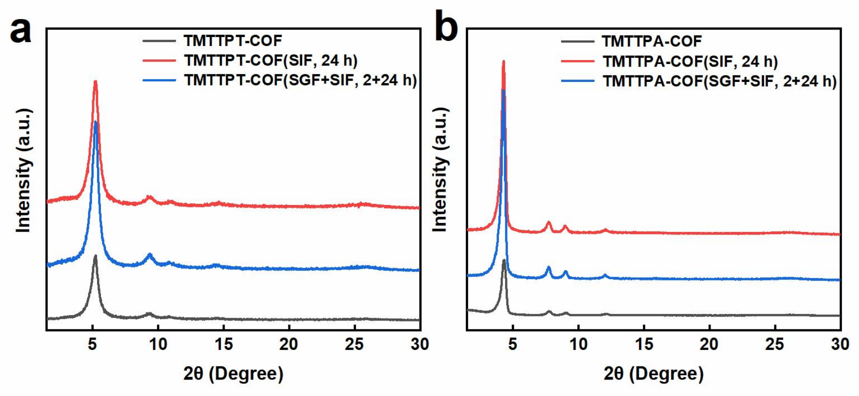

Supplementary Fig. 7 PXRD patterns of TMTTPT-COF (**a**) and TMTTPA-COF (**b**) in the SIF and SGF. (SIF: Simulated intestinal fluid, SGF: Simulated gastric fluid).

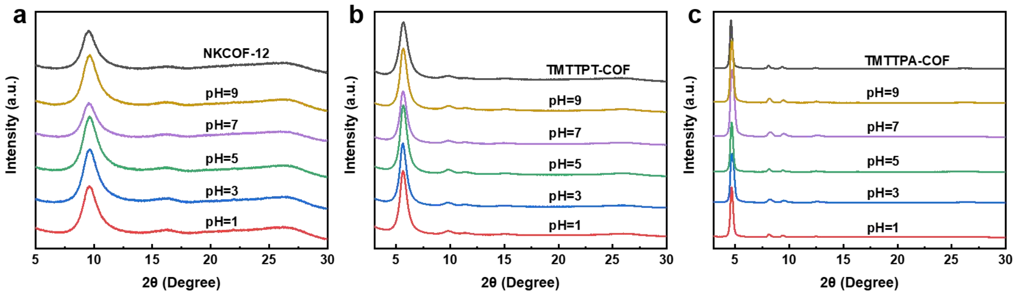

Supplementary Fig. 8 PXRD patterns of NKCOF-12 (**a**), TMTTPT-COF (**b**) and TMTTPA-COF (**c**) after 24 h treatment with aqueous solutions at pH 1, 3, 5, 7, and 9.

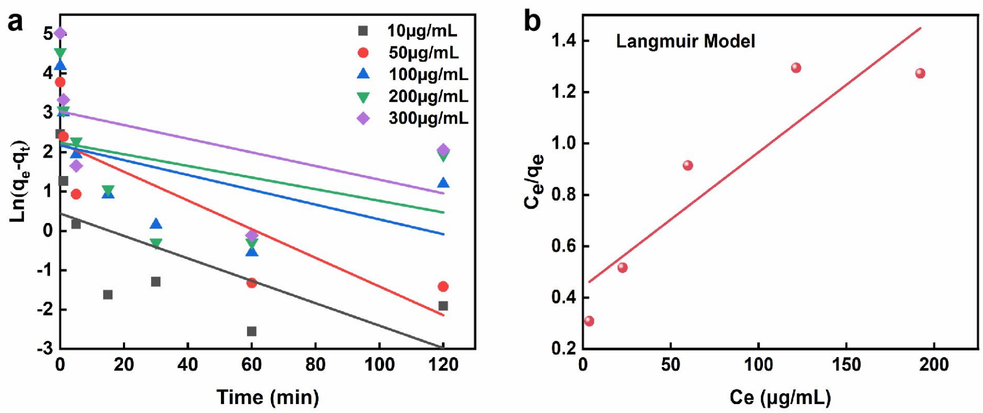

Supplementary Fig. 9 Adsorption experiment of NKCOF-12. a Adsorption kinetic quasi-first-order fitting plots for indole adsorption. **b** Langmuir model fitting plots for indole adsorption isotherms.

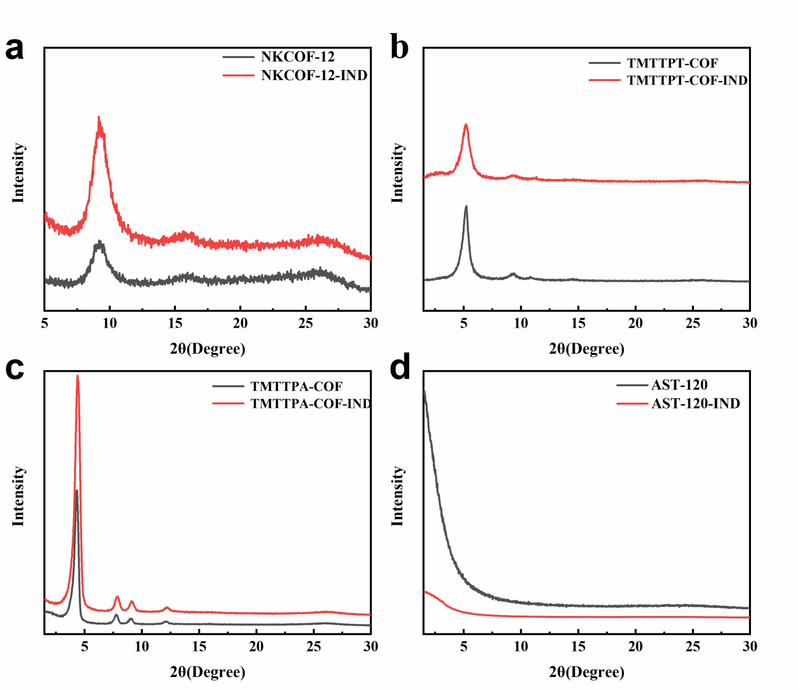

Supplementary Fig. 10 PXRD patterns of NKCOF-12 (**a**), TMTTPT-COF (**b**), TMTTPA-COF (**c**) and AST-120 (**d**) before and after indole adsorption.

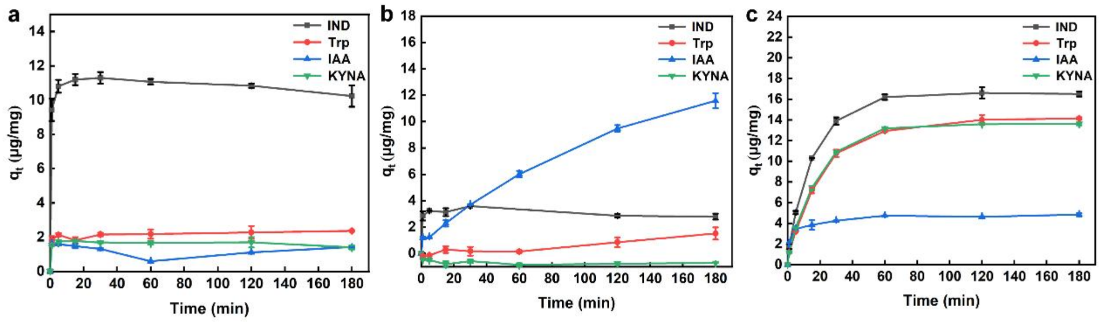

Supplementary Fig. 11 Affinity of TMTTPT-COF (**a**), TMTTPA-COF (**b**) and AST-120 (**c**) for indole in a single solution system. Data were represented as the mean ± SD. The error bars are the standard deviations from three parallel measurements. (IND: indole, IAA: indole-3-acetic acid, Trp: tryptophan, KYNA: kynurenic acid).

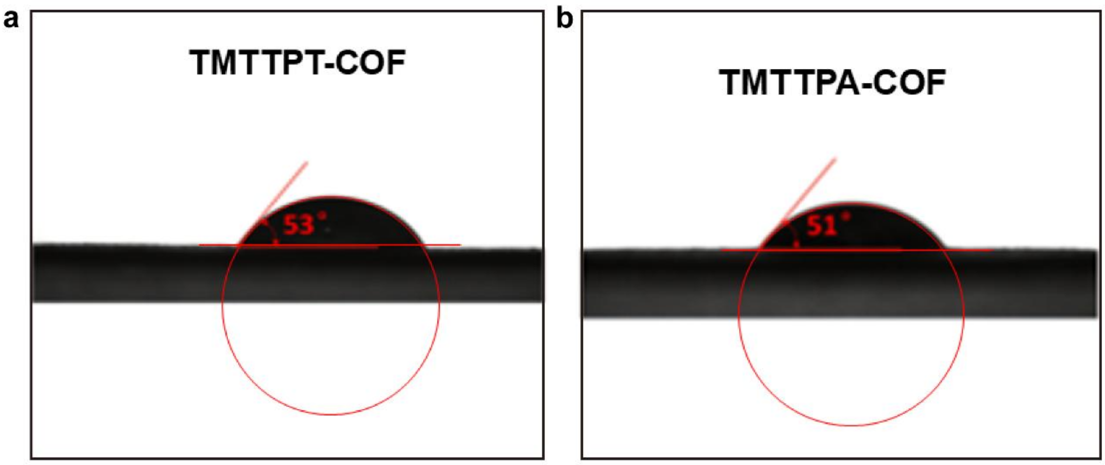

Supplementary Fig. 12 Water contact angles of TMTTPT-COF (**a**) and TMTTPA-COF (**b**).

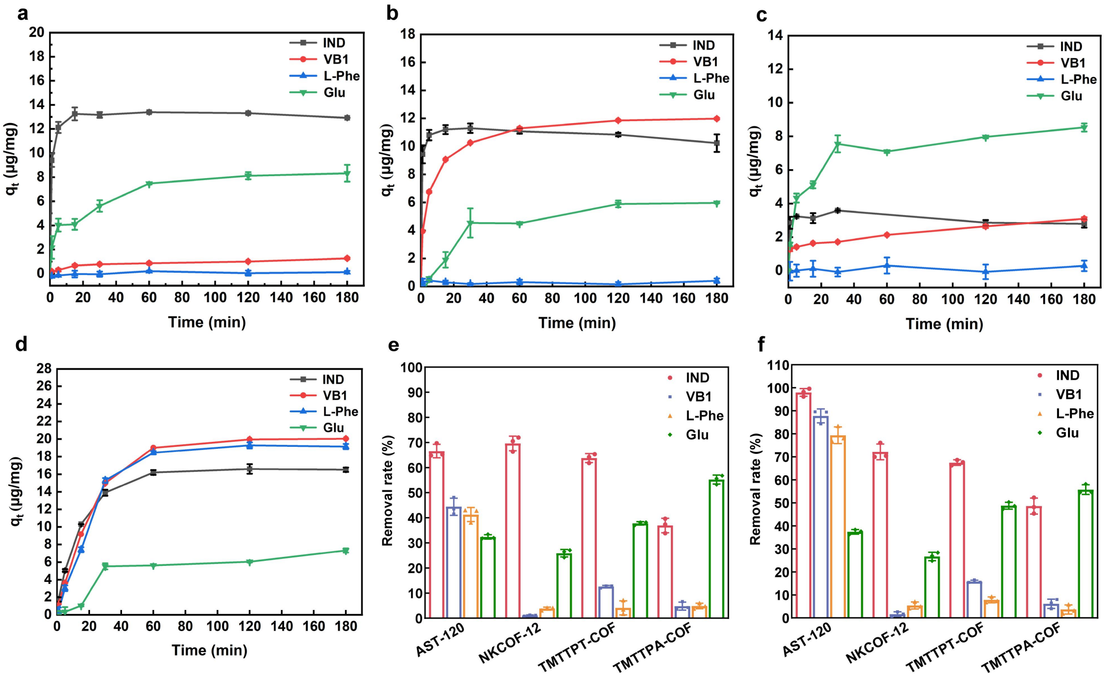

Supplementary Fig. 13 Competitive adsorption of COFs and AST-120 toward indole. Affinity of NKCOF-12 (**a**), TMTTPT-COF (**b**), TMTTPA-COF (**c**) and AST-120 (**d**) for indole in a single solution system, and selective removal rate of adsorbents for indole in a mixture system composed of three nutrients and indole within 15 min (**e**) and 1 h (**f**). Data were presented as the mean ± SD (n = 3). (IND: indole, VB1: vitamin B1, L-Phe: L-phenylalanine, Glu: glucose).

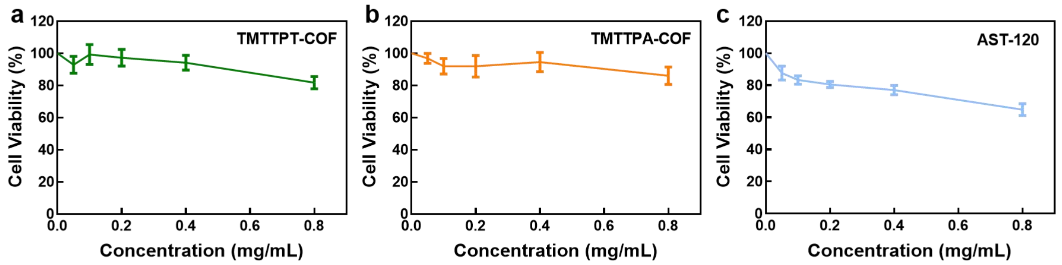

Supplementary Fig. 14 In vitro cytocompatibility experiment. Cell viability of Caco-2 cells after 24 h incubation with different concentrations of TMTTPT-COF (**a**), TMTTPA-COF (**b**) and AST-120 (**c**). Data were presented as the mean ± SD (n = 6).

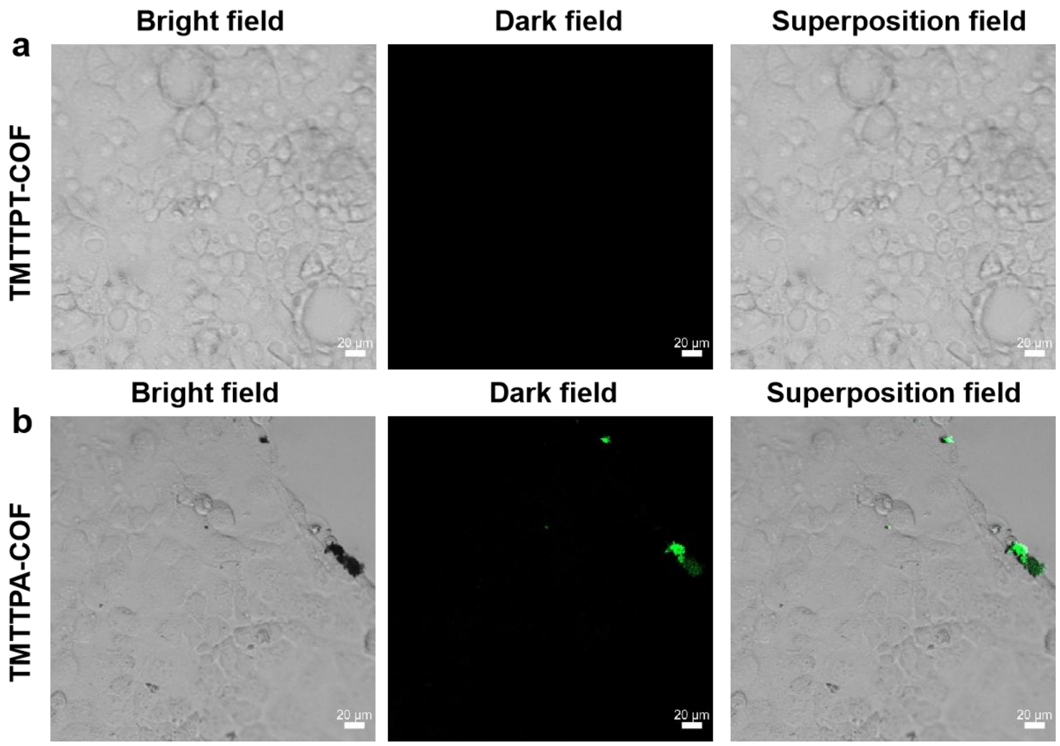

Supplementary Fig. 15 Cellular uptake experiments. Confocal images of Caco-2 cells recorded at 24 h after co-incubation with TMTTPT-COF (**a**) and TMTTPA-COF (**b**). Scale bar = 20 μm. Each experiment was independently repeated three times.

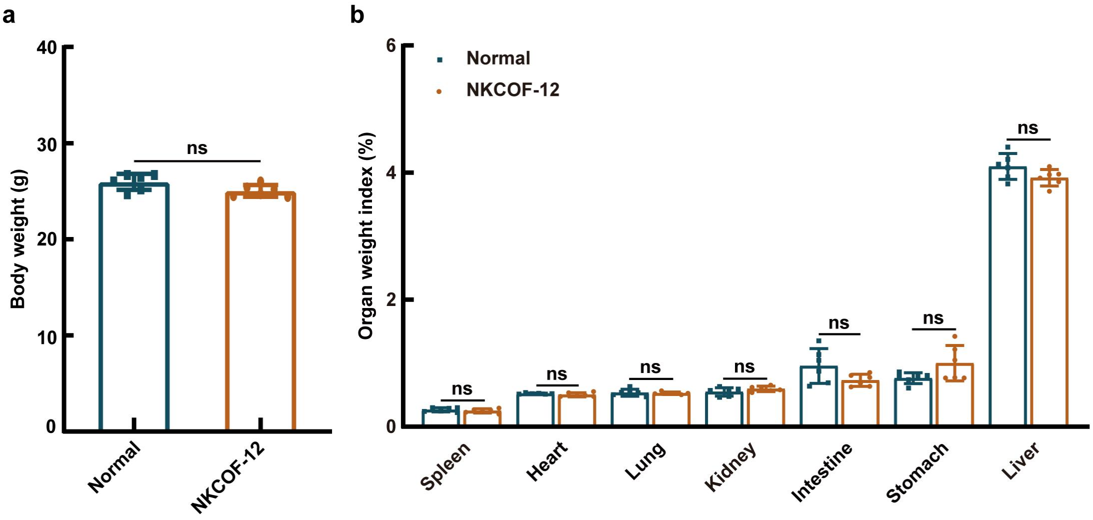

Supplementary Fig. 16 Body weight (**a**) and organ weight index (**b**) of mice of different groups after dosing for 8 weeks. Data were represented as the mean ± SD (*n* = 6). ns: no significance. Statistics were calculated using two-sided independent sample *t*-test.

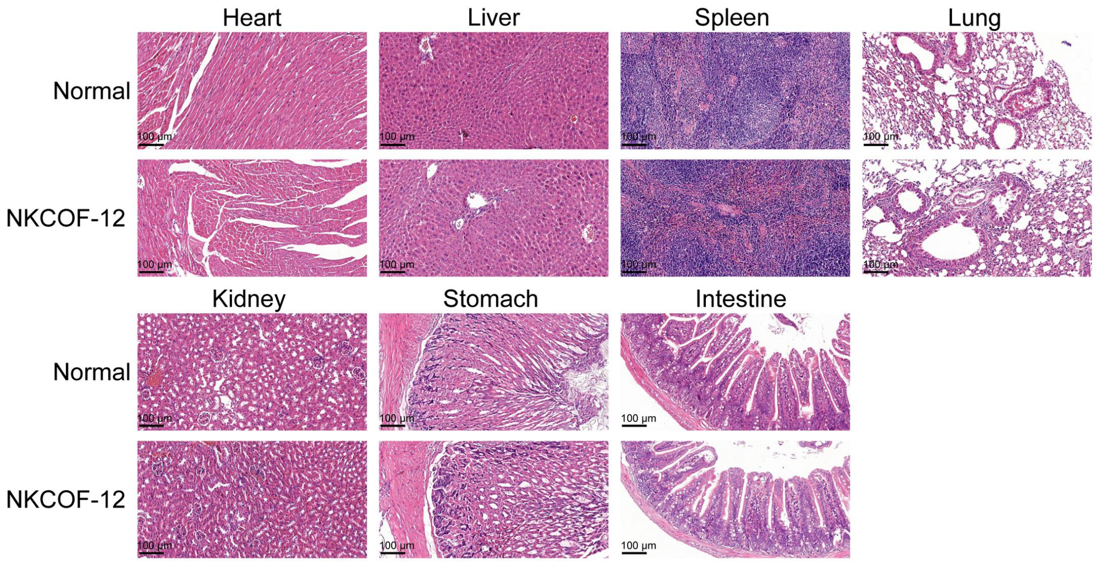

Supplementary Fig. 17 Micrographs of H&E-stained major organs (heart, liver, spleen, lung, kidney, stomach, and intestine) slices collected from mice of different groups. Scale bar = 100 μm. Each experiment was independently repeated three times.

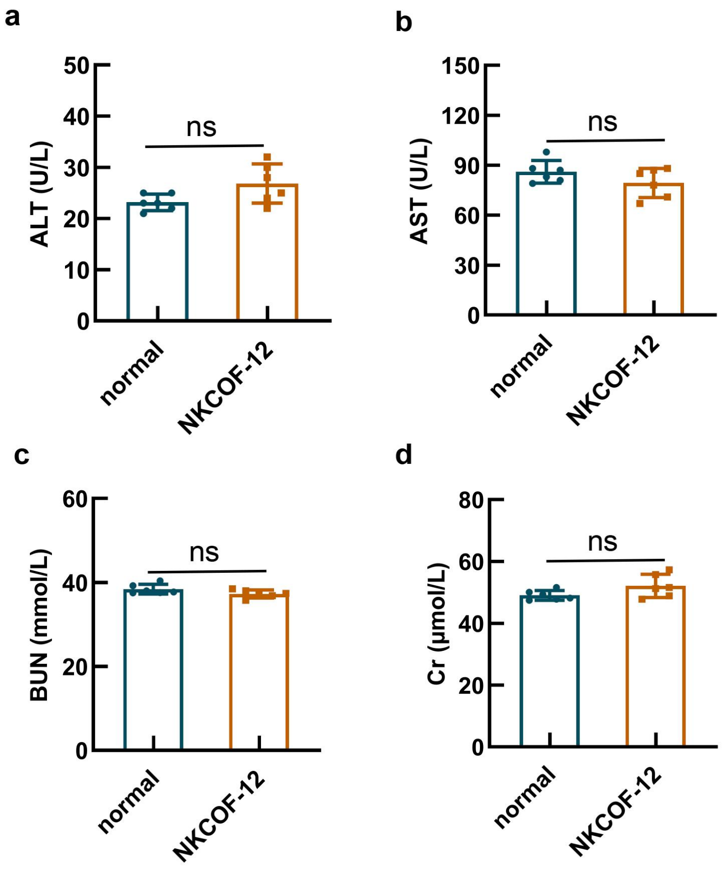

Supplementary Fig. 18 Serum biochemistry examination of the variation in ALT (**a**), AST (**b**), BUN (**c**), and Cr (**d**) after dosing for 8 weeks. Data were represented as the mean ± SD (n = 6). ns: no significance. Statistics were calculated using two-sided independent sample t-test.

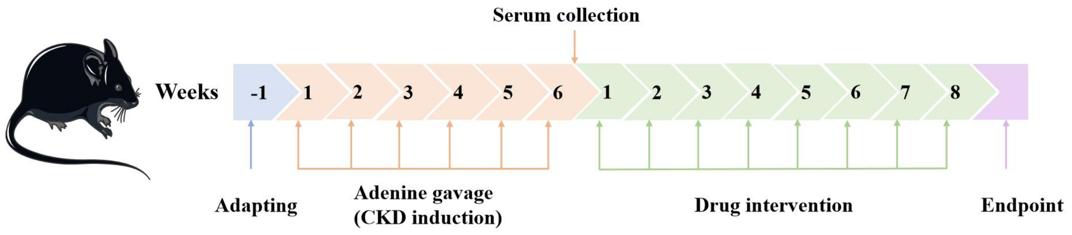

Supplementary Fig. 19 C57BL/6J mice were given diet containing 0.2% adenine for 6 weeks to induce chronic kidney disease. After successfully modeling, the CKD mice in NKCOF-12 and AST-120 groups received oral gavages of NKCOF-12 and AST-120 for 8 weeks, respectively. Untreated CKD mice were used as the model group. All mice were sacrificed after 8 weeks of dosing.

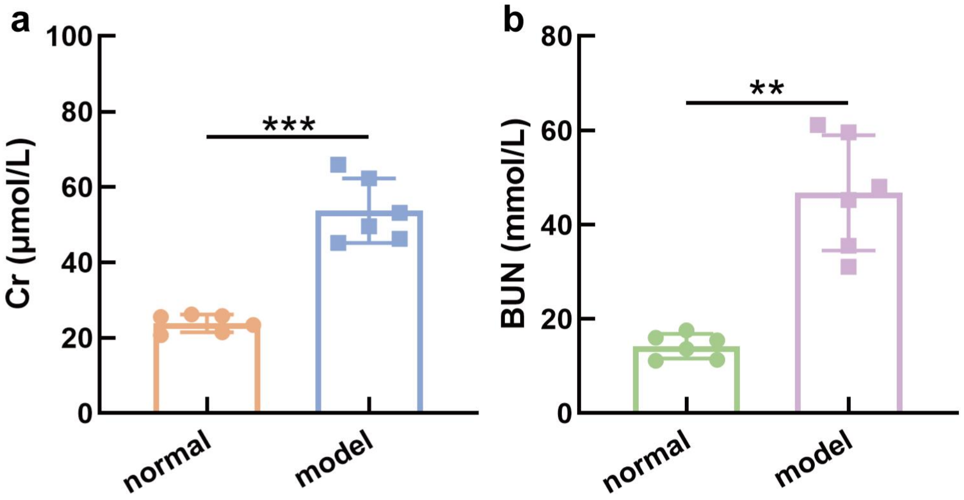

Supplementary Fig. 20 Serum Cr (**a**) and BUN (**b**) levels in the normal and model groups. Data were represented as the mean ± SD (n = 6). *** P = 0.0002, ** P = 0.001007. Statistics were calculated using two-sided independent sample t-test.

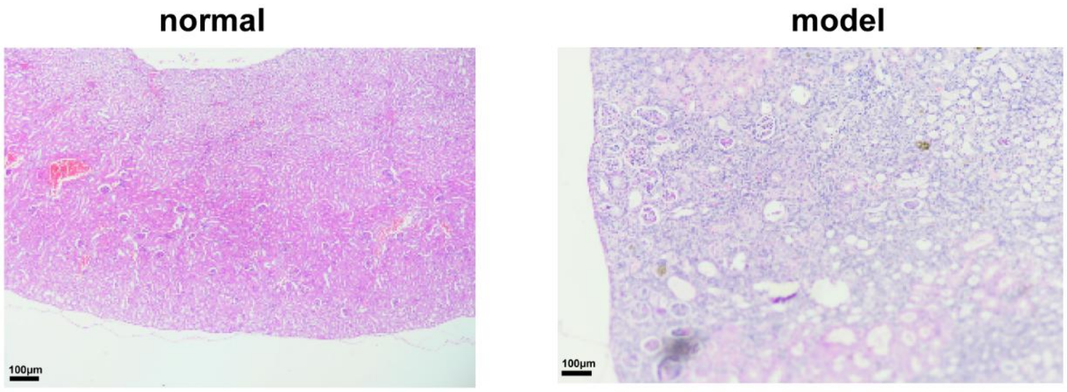

Supplementary Fig. 21 Micrographs of H&E-stained kidney slices from mice of normal and model groups. Scale bar = 100 μm. Each experiment was independently repeated three times.

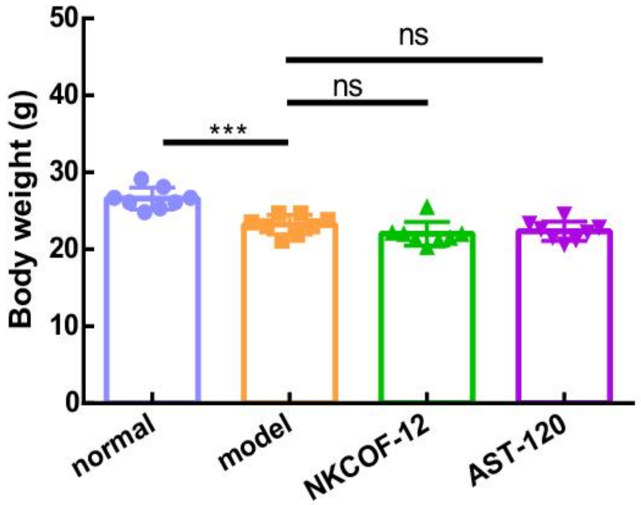

Supplementary Fig. 22 Body weight of mice of different groups after dosing for 8 weeks. Data were represented as the mean ± SD (n = 8). *** P = 0.0002, ns: no significance. Statistics were calculated by one-way ANOVA followed by Tukey's post-test.

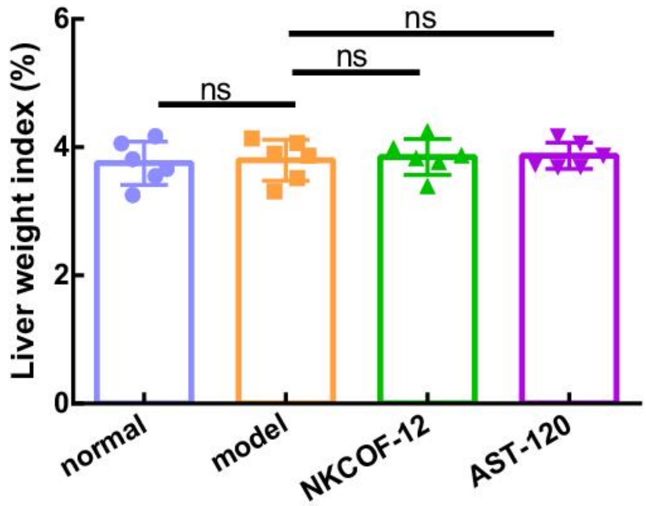

Supplementary Fig. 23 Liver weight index of mice of different groups after dosing for 8 weeks. Data were represented as the mean ± SD (n = 6). ns: no significance. Statistics were calculated by one-way ANOVA followed by Tukey's post-test.

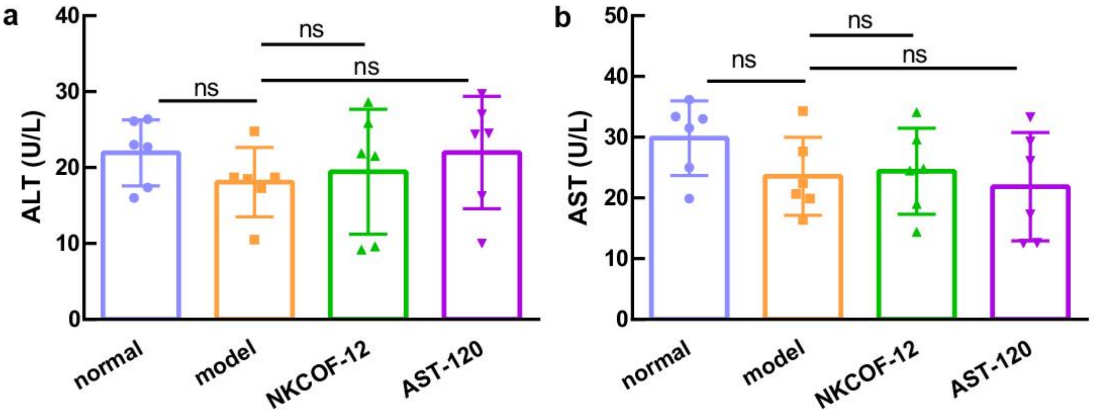

Supplementary Fig. 24 Serum ALT (**a**) and AST (**b**) levels of mice of different groups after dosing for 8 weeks. Data were represented as the mean ± SD (*n* = 6). ns: no significance. Statistics were calculated by one-way ANOVA followed by Tukey's post-test.

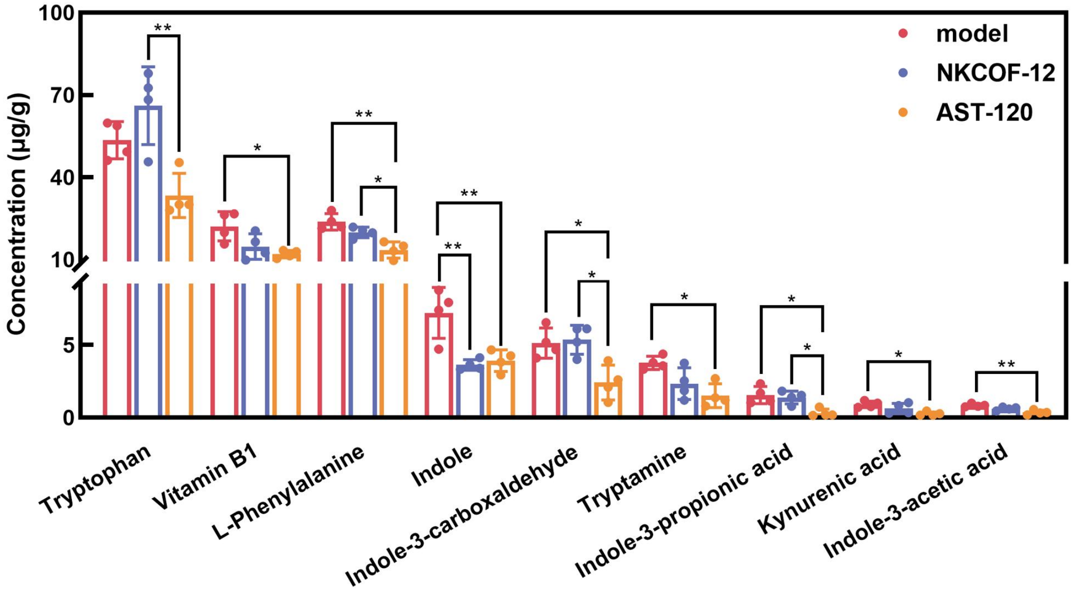

Supplementary Fig. 25 Levels of 9 substances in cecal contents of mice with or without NKCOF-12/AST-120 treatment. UHPLC-QTRAP-MS/MS analysis was utilized to quantitate 9 substances in cecal contents. Data were represented as the mean ± SD (*n* = 4). * $P < 0.05$, ** $P < 0.01$. Statistics were calculated by one-way ANOVA followed by Tukey's post-test. Exact *P* values are given in the Supporting Information file.

Supplementary References

1. Zhang, P., Wang, Z., Yang, Y. et al. Melt polymerization synthesis of a class of robust self-shaped olefin-linked COF foams as high-efficiency separators. *Sci China Chem*. **65**, 1173-1184 (2022).
