# Supplementary material for: Efficient selective removal of uremic toxin precursor by olefin-linked covalent organic frameworks for nephropathy treatment
Source: Nat Commun. 2023 May 16;14:2805. doi: 10.1038/s41467-023-38427-3 (PMC10188479; doi:10.1038/s41467-023-38427-3)
Supplement: Supplementary file 2 — Reporting Summary [file 41467_2023_38427_MOESM2_ESM.pdf]

nature portfolio

Corresponding author(s): Yao Chen

Last updated by author(s): Apr 10, 2023

Reporting Summary

Nature Portfolio wishes to improve the reproducibility of the work that we publish. This form provides structure for consistency and transparency in reporting. For further information on Nature Portfolio policies, see our Editorial Policies and the Editorial Policy Checklist.

Statistics

For all statistical analyses, confirm that the following items are present in the figure legend, table legend, main text, or Methods section.

n/a	Confirmed	
☐	☒	The exact sample size (*n*) for each experimental group/condition, given as a discrete number and unit of measurement
☐	☒	A statement on whether measurements were taken from distinct samples or whether the same sample was measured repeatedly
☐	☒	The statistical test(s) used AND whether they are one- or two-sided<br>*Only common tests should be described solely by name; describe more complex techniques in the Methods section.*
☒	☐	A description of all covariates tested
☒	☐	A description of any assumptions or corrections, such as tests of normality and adjustment for multiple comparisons
☐	☒	A full description of the statistical parameters including central tendency (e.g. means) or other basic estimates (e.g. regression coefficient) AND variation (e.g. standard deviation) or associated estimates of uncertainty (e.g. confidence intervals)
☐	☒	For null hypothesis testing, the test statistic (e.g. *F*, *t*, *r*) with confidence intervals, effect sizes, degrees of freedom and *P* value noted<br>*Give P values as exact values whenever suitable.*
☒	☐	For Bayesian analysis, information on the choice of priors and Markov chain Monte Carlo settings
☒	☐	For hierarchical and complex designs, identification of the appropriate level for tests and full reporting of outcomes
☒	☐	Estimates of effect sizes (e.g. Cohen's *d*, Pearson's *r*), indicating how they were calculated

Our web collection on statistics for biologists contains articles on many of the points above.

Software and code

Policy information about availability of computer code

Data collection	Date were collected with various laboratory instruments and equipments, such as powder X-ray diffraction (PXRD, Rigaku, Japan), scanning electron microscopy (SEM, HITACHI SU3500, Japan), UV-Visible spectrophotometers (UV/vis, HITACHI HU5300, Japan), reverse phase high performance liquid chromatograph (HPLC, Agilent 1260, USA), etc.
Data analysis	Origin 2019, Graph Pad Prism 8.0, IBM SPSS Statistics v21.0.

For manuscripts utilizing custom algorithms or software that are central to the research but not yet described in published literature, software must be made available to editors and reviewers. We strongly encourage code deposition in a community repository (e.g. GitHub). See the Nature Portfolio guidelines for submitting code & software for further information.

Data

Policy information about availability of data

All manuscripts must include a data availability statement. This statement should provide the following information, where applicable:

- Accession codes, unique identifiers, or web links for publicly available datasets
- A description of any restrictions on data availability
- For clinical datasets or third party data, please ensure that the statement adheres to our policy

All data supporting the findings of this study are available within the article, as well as the Supplementary Information file, or available from the authors on request.

Human research participants

Policy information about studies involving human research participants and Sex and Gender in Research.

Reporting on sex and gender	N/A
Population characteristics	N/A
Recruitment	N/A
Ethics oversight	N/A

Note that full information on the approval of the study protocol must also be provided in the manuscript.

Field-specific reporting

Please select the one below that is the best fit for your research. If you are not sure, read the appropriate sections before making your selection.

☒ Life sciences ☐ Behavioural & social sciences ☐ Ecological, evolutionary & environmental sciences

For a reference copy of the document with all sections, see nature.com/documents/nr-reporting-summary-flat.pdf

Life sciences study design

All studies must disclose on these points even when the disclosure is negative.

Sample size	Sample sizes were chosen based on previously published literatures (Materials Science & Engineering C, 2020, 117: 111243; Nature Communications, 2021, 12: 1689) and protocols in the field with a similar setup that showed statistical significance. For each experiment, we have adopted at least n=3 biological replicates to calculate the statistical value of each analysis and the exact sample sizes and statistical data for each experiment are reported in the figure legends.
Data exclusions	No data were excluded from the analyses.
Replication	All experiments were repeated at least three times with reproducibility. All attempts at replication were successful and the exact numbers were shown in the figure legends or tables for all experiments.
Randomization	Mice were randomly assorted to each experimental group when received from the supplier. For all other experiments, samples were also randomly allocated into experimental groups.
Blinding	The investigators were blinded to group allocation during data collection and analysis.

Reporting for specific materials, systems and methods

We require information from authors about some types of materials, experimental systems and methods used in many studies. Here, indicate whether each material, system or method listed is relevant to your study. If you are not sure if a list item applies to your research, read the appropriate section before selecting a response.

Materials & experimental systems

n/a	Involved in the study	
☒	☐	Antibodies
☐	☒	Eukaryotic cell lines
☒	☐	Palaeontology and archaeology
☐	☒	Animals and other organisms
☒	☐	Clinical data
☒	☐	Dual use research of concern

Methods

n/a	Involved in the study	
☒	☐	ChIP-seq
☒	☐	Flow cytometry
☒	☐	MRI-based neuroimaging

Eukaryotic cell lines

Policy information about cell lines and Sex and Gender in Research

Cell line source(s)	Caco-2 cell line was purchased from Procell Life Science & Technology Co., Ltd. (Wuhan, China).

Authentication	Authentication was performed by analysis of morphology.
Mycoplasma contamination	The cell line tested negative for mycoplasma contamination.
Commonly misidentified lines (See ICLAC register)	No commonly misidentified lines were used in this study.

Animals and other research organisms

Policy information about studies involving animals; ARRIVE guidelines recommended for reporting animal research, and Sex and Gender in Research

Laboratory animals	Six-week-old male C57BL/6J mice were obtained from Beijing Military Medical Science Academy of the PLA (Beijing, China) in this study. They were adaptively housed in the SPF facility for one week under a 12 h light/dark cycle, with free access to water and Sterilized AIN93G pellets (Beijing HFK Biotechnology Co., Ltd., China). Ambient temperature is about 25 °C and the relative humidity is 55-60%.
Wild animals	This study did not involve use of wild animals.
Reporting on sex	Sex was not considered in this study design.
Field-collected samples	This study did not involve samples collected from the field.
Ethics oversight	All experiments involving animals were approved by the Care and Use Committee of the Animal Experiment Center of Tianjin University of Traditional Chinese Medicine, Tianjin, China.

Note that full information on the approval of the study protocol must also be provided in the manuscript.
